# Adaptive Cellular Responsiveness to Electrical Stimulation at the Skin‐Electrode Interface

**DOI:** 10.1002/advs.78525

**Published:** 2026-10-27

**Authors:** Yeon Jae Chang, Soon Jin Choi, Khandoker Asiqur Rahaman, Tae Kyoung Lee, Gwangbin Lee, Jaeyeon Jun, Hyeon‐Jeong Lee, Hophil Min, Hojeong Jeon, Jung Keun Hyun, Hyung‐Seop Han, Jimin Park

**Affiliations:** ^1^ Department of Chemical and Biomolecular Engineering Korea Advanced Institute of Science and Technology (KAIST) Daejeon South Korea; ^2^ Center for Biomaterials Korea Institute of Science and Technology (KIST) Seoul South Korea; ^3^ KU‐KIST Graduate School of Converging Science and Technology Korea University Seoul South Korea; ^4^ Interdisciplinary Research Center for Biosystems and Machines King Fahd University of Petroleum & Minerals Dhahran Saudi Arabia; ^5^ Defense of Forensic Service, Criminal Investigation Command Ministry of National Defense Seoul South Korea; ^6^ Doping Control Center Korea Institute of Science and Technology (KIST) Seoul South Korea; ^7^ College of Medicine Ajou University Suwon South Korea

**Keywords:** bioelectronic interface, calcium signaling, electrical stimulation, regenerative response, voltage‐gated calcium channels, wound healing

## Abstract

Wearable electrical stimulation (ES) devices are emerging platforms for wound healing, where cellular responses at the skin‐electrode interface critically influence therapeutic outcomes. Here, we demonstrate that skin cells dynamically adapt to sustained ES by upregulating voltage‐gated calcium channels (VGCCs) and enhancing their sensitivity to electrical cues. Using a transparent indium tin oxide (ITO)‐based bioelectronic platform, we find that ES‐induced upregulation of the L‐type VGCC Ca_V_1.3 amplifies intracellular calcium (Ca^2+^) influx during subsequent stimulation. ES delivered through a skin‐conformal ITO‐based wearable device similarly increases Ca_V_1.3 expression in mouse wound tissue, accompanied by accelerated wound healing, enhanced collagen deposition, vascular remodeling, and changes in the CD206/iNOS macrophage‐associated marker balance. Pharmacological inhibition of L‐type VGCCs with nifedipine suppresses Ca_V_1.3 upregulation and attenuates these regenerative responses, supporting a functional contribution of VGCC‐mediated Ca^2+^ signaling to ES‐assisted wound repair. Human skin cells further exhibit enhanced Ca^2+^ responsiveness and coordinated transcriptional and proteomic remodeling following sustained ES. These findings identify adaptive VGCC‐associated Ca^2+^ signaling as a key cellular mechanism underlying sustained ES responses at the bioelectronic interface.

## Graphical abstract

This study presents an adaptive bioelectronic response in which sustained electrical stimulation (ES) upregulates voltage‐gated calcium channels (VGCCs) and enhances cellular sensitivity to subsequent ES at the skin‐device interface. VGCC‐associated adaptive sensitization promotes regenerative wound‐healing responses in vivo, including accelerated wound closure, enhanced angiogenesis, and regulated CD206/iNOS macrophage‐associated marker balance. Proteomic analysis of human skin cells further supports ES‐induced molecular remodeling associated with regenerative cellular responses.

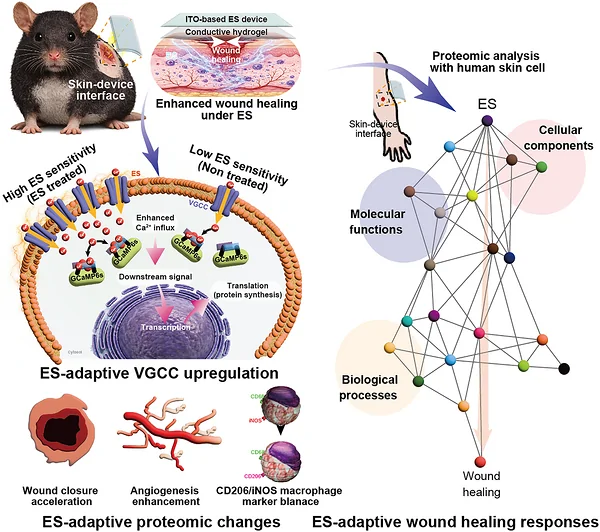

## 1 Introduction

Wearable electrical stimulation (ES) devices have emerged as a promising strategy for accelerating wound healing by delivering localized electrical cues directly to injured skin [1, 2, 3, 4] Their therapeutic effects have been associated with enhanced cell proliferation, migration, angiogenesis, and tissue remodeling, leading to growing interest in bioelectronics for wound care [5, 6, 7]. As these systems operate through direct contact with damaged tissue, the skin‐device interface becomes the primary site at which applied electrical inputs are first received and translated into biological responses [8, 9, 10]. Defining how skin cells interpret and adapt to ES at this interface is therefore essential for understanding the ES‐assisted wound repair process [11, 12, 13].

Voltage‐gated ion channels (VGICs) in skin cells are key mediators of cellular responses to ES at the skin‐device interface because they directly couple extracellular electrical cues to intracellular signaling [14, 15]. Applied electric fields (EFs) can alter membrane potential, leading to the activation or deactivation of VGICs and consequent changes in ion flux that initiate intracellular biochemical signaling [16]. Among the diversity of VGICs, voltage‐gated calcium channels (VGCCs) are of particular interest because they directly regulate calcium (Ca^2+^) influx, a central signal that controls proliferation, survival, migration, and repair‐associated gene regulation [17]. Ca^2+^ signaling is also a major upstream regulator of pathways broadly implicated in wound repair, including phosphoinositide 3‐kinase (*PI3K*)‐*AKT*‐mechanistic target of rapamycin (*mTOR*), rapidly accelerated fibrosarcoma (*Raf*)‐mitogen‐activated protein kinase kinase (*MEK*)‐extracellular signal‐regulated kinase (*ERK*), and glycogen synthase kinase 3β (*GSK3β*)‐associated signaling [18, 19].

While these insights have advanced our understanding of VGCC‐mediated signaling upon ES, most prior studies have focused on downstream events rather than on how skin cells adapt to ES [20, 21]. Since ES is typically applied continuously during wound treatment, the ability of skin cells to dynamically adapt to electrical cues may represent an important determinant of therapeutic outcome [22]. However, it remains unclear whether ES induces dynamic remodeling of VGCC availability, distribution, or functional properties, or more broadly reshapes Ca^2+^‐mediated signaling pathways at the skin‐device interface [23, 24]. Understanding these adaptive processes could therefore provide mechanistic insight into how ES promotes wound healing [25].

Here, we demonstrated how sustained ES at the skin‐bioelectronic interface reshapes the responsiveness of skin cells to subsequent ES and how this adaptive response contributes to wound repair. Using a transparent indium tin oxide (ITO) thin‐film bioelectronic interface, we show that sustained ES enhances VGCC‐associated responsiveness in mouse skin cells, amplifying intracellular Ca^2+^ influx during subsequent stimulation, as identified by live‐cell Ca^2+^ imaging. These adaptive cellular responses are further recapitulated in mouse wound tissue in vivo. Based on finite element analysis (FEA), we translated the effective in vitro electrical environment into a wound healing setting, where EF‐matched ES increases VGCC expression and promotes regenerative wound healing‐related cellular behaviors, including accelerated wound closure, angiogenic remodeling, and reparative macrophage‐associated responses. Finally, experiments using primary human skin cells also support the broader relevance of this VGCC‐centered adaptive response at the skin‐bioelectronic interface and its associated molecular remodeling during sustained ES (Figure 1).

**FIGURE 1:**
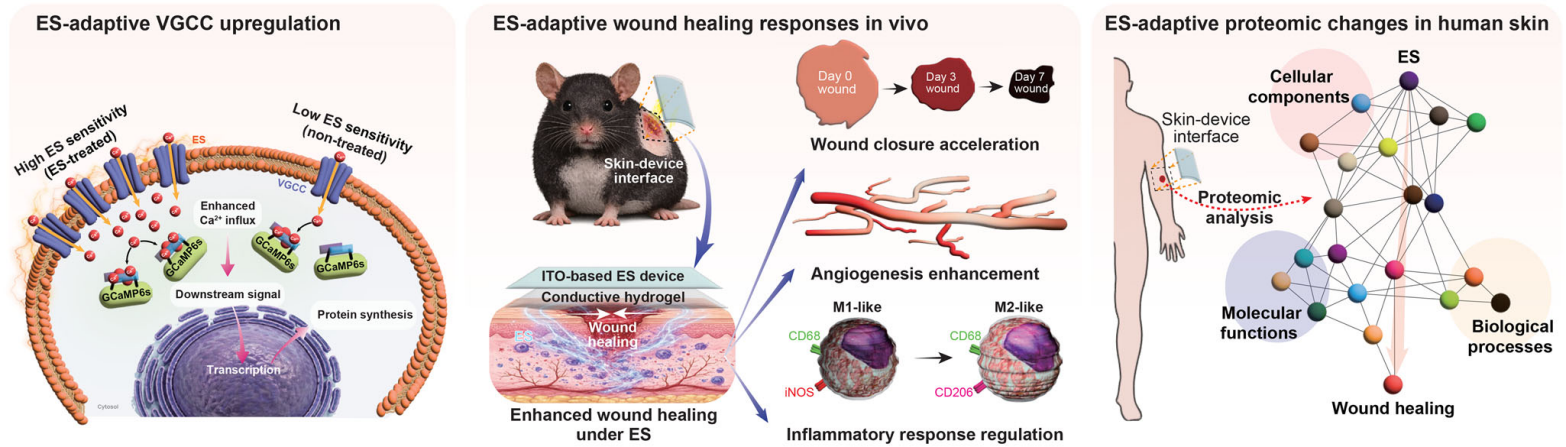
Schematic overview of ES‐induced adaptive cellular responsiveness at the skin‐device interface. Sustained ES at the skin‐device interface induces VGCC upregulation and enhances Ca^2+^ influx during subsequent stimulation, resulting in increased cellular sensitivity to ES. In a mouse wound model, this adaptive VGCC‐associated response is linked to accelerated wound closure, enhanced angiogenesis, and regulated inflammatory responses within the wound tissue. Human skin‐cell proteomic analysis further shows ES‐induced molecular remodeling associated with wound‐healing‐related cellular responses.

## 2 Results

### 2.1 ES‐Induced Enhancement of VGCC Expression and Electrical Responsiveness in Skin Cells

To investigate cellular adaptation to ES at the skin‐ES device interface, we established a model platform comprising a transparent ITO thin‐film electrode together with murine melanoma B16F10 cells. The ITO thin‐film electrode was employed because its transparent, biocompatible, and conductive properties uniquely enable real‐time optical monitoring of adaptive cellular responses to ES at the cell‐electrode interface [26]. B16F10 cells were employed as electrically responsive skin‐cell models because they express multiple VGICs, including voltage‐gated sodium (Na^+^), potassium (K^+^), and Ca^2+^ channels [27]. In addition, their robust proliferative capacity and high transfection efficiency with genetically encoded Ca^2+^ indicators made them well‐suited for real‐time imaging of intracellular bio‐signaling dynamics during ES [28].

B16F10 cells were cultured on the ITO thin‐film electrode and exposed to ES across an applied voltage range of 0.00–1.00 V for 24 h. Voltages above 1.00 V were not evaluated, as higher potentials may induce Faradaic reactions, including water electrolysis, local pH perturbations, and reactive oxygen species generation, which could confound the interpretation of electrically induced cellular responses [29, 30]. Among the tested conditions, 0.50 V elicited the most robust increase in cellular proliferation, a key process in wound healing, without inducing observable cytotoxicity (Figure S1), and was therefore selected for subsequent experiments.

To investigate ES‐mediated changes in B16F10 cells, we first analyzed their transcriptional state following ES at 0.50 V for 24 h, using non‐stimulated cells cultured for the same duration as controls. We profiled a targeted panel of 69 genes spanning VGICs, wound healing‐associated signaling pathways, and apoptosis. Among the VGIC genes examined, L‐type VGCC‐associated genes, including *CACNA1D* (Ca_V_1.3), *CACNA1C* (Ca_V_1.2), and *CACNA1E* (Ca_V_2.3), showed a marked increase after ES, whereas voltage‐gated Na^+^ and K^+^ channel genes, including *SCN1A* (Na_V_1.1), *SCN4A* (Na_V_1.4), *SCN8A* (Na_V_1.6), and *KCNA10* (K_V_1.8), exhibited comparatively minor changes (Figure 2a). Volcano plot analysis further identified *CACNA1D* as the most prominently upregulated VGCC‐associated transcript (Figure 2b). In parallel, genes associated with wound healing‐related signaling, including *RAS*, *Raf*, *MEK*, *mTOR*, and *β‐catenin*, were also upregulated (Figure 2a,b), consistent with the enhanced proliferation observed at 0.50 V (Figure S1). In contrast, apoptosis‐related genes, including *Caspase9*, *Bax*, and *Bcl‐2*, did not show significant changes under this condition (Figure 2a).

**FIGURE 2:**
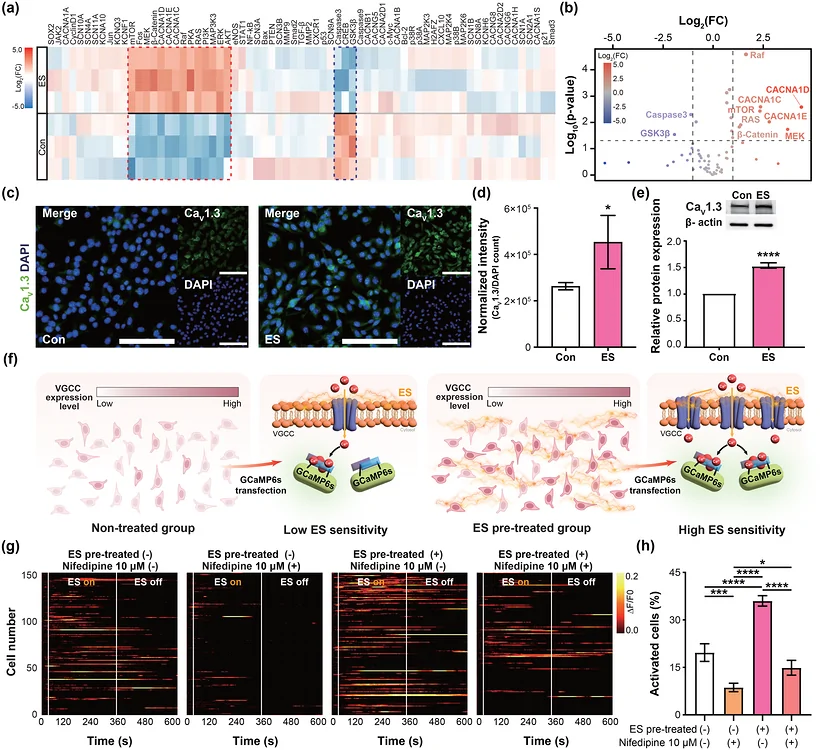
ES‐induced VGCC upregulation and enhanced cellular sensitivity to ES in vitro. (a) Heatmap showing differential expression of 69 target genes (Log_2_fold change, Log_2_FC) between B16F10 cells exposed to ES at 0.50 V for 24 h (ES) and non‐stimulated controls (Con). Red and blue colors indicate upregulation and downregulation, respectively. Genes showing statistically significant differences between the two groups, as determined by *t*‐test (*n*  =  3 per group, all *p*‐values in Table S1), are highlighted with dashed boxes (red: upregulated, blue: downregulated). (b) Volcano plot of transcriptomic changes between ES and Con groups. Key regulated genes are labeled, including *CACNA1D* (Ca_V_1.3), *CACNA1C* (Ca_V_1.2), *CACNA1E* (Ca_V_2.3), *Raf*, *mTOR*, *RAS*, *MEK*, *β‐catenin*, *Caspase3*, and *GSK3β*. (c) Representative ICC images of Ca_V_1.3 (green) with DAPI nuclear staining (blue) in Con and ES groups. Merged images and single‐channel views are shown (scale bars, 200 µm). (d) Quantification of Ca_V_1.3 fluorescence intensity normalized to DAPI‐positive cell count (Ca_V_1.3/DAPI count). ES significantly increased Ca_V_1.3 expression compared with Con, confirmed by t‐test (*n  =  *3 per group, *p*‐value = 0.0471, *t* = 2.835, df = 4). (e) Western blot analysis of Ca_V_1.3 protein expression in Con and ES groups, with β‐actin as a loading control. The upper panel displays a representative Western blot image showing Ca_V_1.3 in Con and ES. The bar graph shows densitometric quantification of relative protein expression. ES significantly increased Ca_V_1.3 protein levels, confirmed by a *t*‐test (*n  =  *3 per group, *p*‐value < 0.0001, t = 16.85, df = 4). (f) Schematic illustration of ES‐induced VGCC upregulation and enhanced Ca^2+^ responses to subsequent ES. Cells without prior ES exhibit limited Ca^2+^ influx, whereas ES pre‐treated cells show enhanced GCaMP6s signals upon subsequent stimulation. (g) Heatmaps showing individual GCaMP6s fluorescence changes (ΔF/F_0_) before, during, and after ES in 150 B16F10 cells, with 50 cells randomly selected from each of three independent biological experiments. ES pre‐treated denotes prior exposure to ES at 0.50 V for 24 h before Ca^2+^ imaging, whereas nifedipine denotes treatment with 10 µM nifedipine. During Ca^2+^ imaging, ES was applied from 30 to 330 s (white lines). (h) Quantification of the percentage of activated cells during subsequent ES. ES pre‐treated cells showed a significantly greater proportion of activated cells upon subsequent stimulation, whereas nifedipine attenuated this effect. For each condition, 50 cells were randomly selected from each of three independent biological experiments (150 cells in total), and the percentage of activated cells was calculated separately for each experiment. Statistical analysis was performed using these independent replicate‐level measurements (*n* = 3 per condition) by one‐way analysis of variance (ANOVA) followed by Tukey's multiple comparison test. All *p*‐values are listed in Table S2. Data in Figure 2d,e,h are presented as mean ± standard error of the mean (s.e.m.). Statistical significance is indicated as *p < 0.05, ***p < 0.001, ****p < 0.0001.

To account for multiple comparisons across the 69‐gene panel, raw *p*‐values were adjusted using the two‐stage Benjamini‐Krieger‐Yekutieli (BKY) procedure, with the false discovery rate (FDR) < 10% [31, 32, 33]. Of the 19 genes showing nominal significance before correction, 18 remained significant after FDR correction, whereas *KCNA10* did not meet the BKY false positive threshold (Figure S2). Importantly, the ES‐induced changes in VGCC‐associated genes remained significant after multiple‐testing correction, further highlighting VGCCs as key components of the cellular response to ES delivered through the skin‐device interface.

Consistent with these transcriptional findings, ES was accompanied by increased Ca_V_1.3 protein expression in B16F10 cells. Immunocytochemistry (ICC) revealed stronger Ca_V_1.3 fluorescence in ES‐treated cells compared with non‐stimulated controls (Figure 2c). Quantitative analysis of normalized Ca_V_1.3 intensity (Ca_V_1.3/DAPI count) showed a 72.3 ± 26.0% increase in the ES‐treated group relative to controls (Figure 2d). Western blot analysis further confirmed elevated Ca_V_1.3 protein levels in the ES‐treated group, with an approximately 1.5‐fold increase relative to controls (Figure 2e).

To determine whether ES‐induced Ca_V_1.3 upregulation also occurs in wound‐relevant cell types, we examined Ca_V_1.3 expression in human umbilical vein endothelial cells (HUVECs) and National Institutes of Health / 3‐day transfer, inoculum 3 × 10^5^ (NIH3T3) fibroblasts using ICC. HUVECs were included as an endothelial cell model relevant to vascular remodeling, whereas NIH3T3 cells were examined as mesenchymal cell models associated with tissue repair and remodeling. Consistent with the findings in B16F10 cells, ES increased Ca_V_1.3 fluorescence in both cell types compared with their respective non‐stimulated controls (Figures S3 and S4). The consistent increase in Ca_V_1.3 expression observed in HUVECs and NIH3T3 fibroblasts indicates that the ES‐associated Ca_V_1.3 response is not restricted to B16F10 cells but also extends to other wound‐relevant cellular models.

To determine whether adaptive increases in Ca_V_1.3 expression following ES alter cellular Ca^2+^ responsiveness to subsequent stimulation observed in B16F10, we performed live‐cell Ca^2+^ imaging. Owing to the optical transparency of the ITO thin‐film electrode, real‐time monitoring of intracellular Ca^2+^ dynamics at the cell‐electrode interface was feasible. B16F10 cells pre‐treated with ES at 0.50 V for 24 h, as well as cells without prior ES exposure, were transfected with a plasmid encoding the Ca^2+^ indicator GCaMP6s [34]. GCaMP6s fluorescence changes (ΔF/F_0_, where F_0_ represents the baseline fluorescence during the initial 30 s of recording) were then monitored as a readout of intracellular Ca^2+^ influx before, during, and after subsequent ES at 0.50 V (Figure 2f). For quantitative analyses, 150 cells randomly selected from three independent experiments were analyzed, and ΔF/F_0_ traces of individual cells over time were visualized as heatmaps (Figure 2g).

Consistent with the increased Ca_V_1.3 expression, cells pre‐treated with ES for 24 h exhibited enhanced Ca^2+^ responsiveness during subsequent stimulation, as evidenced by a significantly higher fraction of activated cells (36.0 ± 0.95%) compared with cells without prior ES exposure (19.7 ± 1.61%) (Figure 2g,h). Here, cells showing a GCaMP6s fluorescence increase (ΔF/F_0_) greater than 5% during the subsequent ES were defined as activated cells. Pharmacological inhibition with nifedipine, an L‐type VGCC blocker, significantly reduced the activated‐cell fraction in cells previously exposed to ES from 36.0 ± 0.95% to 14.9 ± 1.35%. Similarly, nifedipine treatment decreased ES‐mediated Ca^2+^ responses in cells without prior ES exposure (Figure 2h). In addition to suppressing Ca^2+^ responsiveness, nifedipine attenuated the ES‐induced increase in B16F10 cell proliferation by approximately 27.8 ± 4.15% (Figure S5), indicating that enhanced VGCC‐dependent Ca^2+^ signaling is functionally linked to increased proliferative behavior. Together, these findings indicate that ES induces adaptive VGCC upregulation and sensitizes skin‐associated cells to subsequent ES.

Overall, these findings demonstrate that ES reshapes both the molecular and functional state of skin cells at the skin‐device interface. Sustained ES increases VGCC expression, particularly Ca_V_1.3, and this change is accompanied by enhanced Ca^2+^ responses upon subsequent ES and increased proliferation in B16F10. Similar increases in Ca_V_1.3 expression were also observed in endothelial and fibroblast cell models, indicating that this molecular response is not restricted to B16F10 cells. In this context, VGCC upregulation constitutes a key adaptive mechanism by which skin cells dynamically respond to ES.

### 2.2 ES‐Adaptive VGCC Upregulation and Accelerated Wound Closure In Vivo

We next investigated whether the adaptive cellular responses to ES observed in vitro could also be recapitulated in vivo during skin wound healing (Figure 3a). Our previous study identified EF as a key determinant of ES‐mediated cellular responses, emphasizing the importance of matching the effective EF across experimental systems [26]. Because the effective EF experienced by cells is strongly influenced by tissue geometry and bioelectrical properties, directly applying the in vitro voltage condition to skin tissue was not appropriate.

**FIGURE 3:**
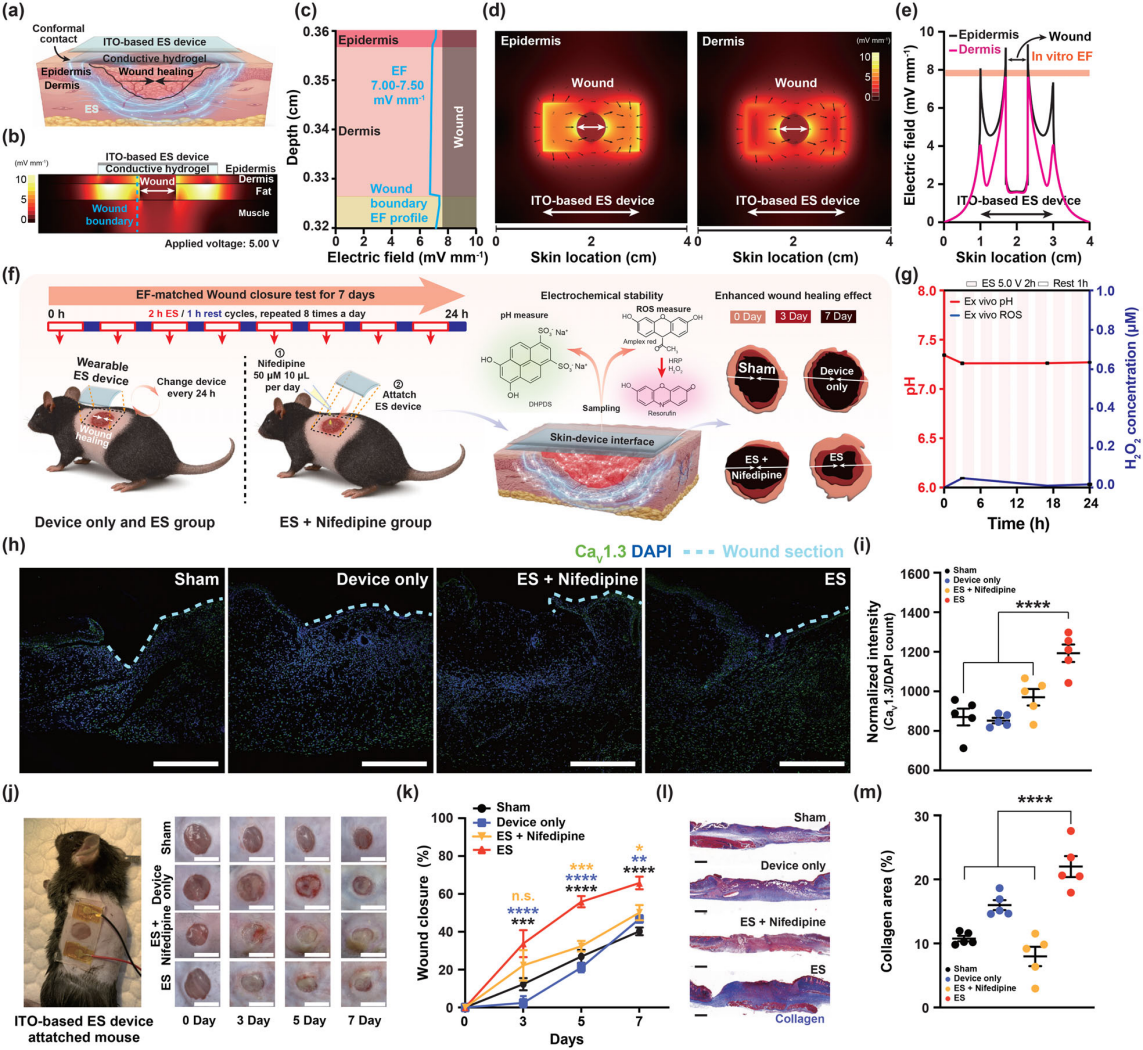
VGCC upregulation and accelerated wound healing promoted by ES in vivo. (a) Schematic of the ITO thin‐film‐based ES device with a conductive hydrogel interface conformally attached over a dorsal full‐thickness skin wound. (b) Calculated EF profiles across the full thickness of mouse skin from epidermis to muscle at 5.00 V. (c) Calculated cross‐sectional EF profiles at 5.00 V, showing that an approximately 7.00‐7.50 mV mm^−1^ EF is generated across the epidermal and dermal regions at the wound boundary. The position of the wound boundary is indicated by the sky‐blue dashed line in (b). (d, e) Calculated in‐plane EF maps of epidermis and dermis regions (d) and corresponding lateral EF profiles within epidermal and dermal layers (e). All parameters used in the FEA simulation are listed in Table S3. (f) Schematic illustration of the in vivo wound‐healing protocol and electrochemical stability assessment under the EF‐matched stimulation condition. The ES device was replaced every 24 h, and 5.00 V ES was delivered using repeated 2 h ES/1 h rest cycles for 7 days. For the ES + Nifedipine group, 10 µL of 50 µM nifedipine was locally applied to the wound before each daily device attachment. Electrochemical stability at the skin‐device interface was evaluated under the same cyclic stimulation condition by monitoring local pH and H_2_O_2_ generation in an ex vivo wound model. (g) Changes in pH and H_2_O_2_ concentration at the ex vivo skin‐device interface during 24 h of 5.00 V ES using repeated 2 h ES/1 h rest cycles. (h) Representative immunofluorescence images of wound tissue sections collected on day 7 from sham, device only, ES + Nifedipine, and ES‐treated groups stained for Ca_V_1.3 (green) and DAPI (blue). The light‐blue dashed curved line indicates the wound region (scale bar =  400 µm, all single‐channel immunofluorescence images are displayed in Figure S16). (i) Quantification of normalized Ca_V_1.3 fluorescence intensity relative to DAPI count in collected wound tissue sections (*n*  =  5 per group). Statistical analyses were performed using one‐way ANOVA and Tukey's multiple comparison test, and all *p*‐values are listed in Table S4. (j) Representative photograph of a mouse with the ES device attached (left) and serial macroscopic images of wound appearance from day 0 to day 7 in the sham, device only, ES + Nifedipine, and ES‐treated groups (right) (scale bar =  5 mm). (k) Wound closure fraction over time, demonstrating accelerated wound closure in the ES‐treated group compared with sham, device only, and ES + Nifedipine groups, as analyzed by two‐way ANOVA and Tukey's multiple comparison test (*n*  =  5 per group, all *p*‐values are listed in Table S5). (l) Representative MT‐stained histological images of wound tissues collected on day 7 (scale bar =  500 µm). (m) Quantitative analysis of the collagen‐stained area (blue) within the wound region. Statistical analysis was performed using one‐way ANOVA followed by Tukey's multiple‐comparison test (*n*  =  5 per group). All *p*‐values are listed in Table S6. For Figure 3i,k,m, results are presented as mean ± s.e.m. Statistical significance is indicated as *p < 0.05, **p < 0.01, ***p < 0.001, ****p < 0.0001.

To define the target EF for in vivo translation, an FEA model was first constructed for the in vitro configuration used in the cell experiments, including the ITO thin‐film electrode, culture medium, and an adherent cell layer at the electrode‐medium interface. Under an applied voltage of 0.50 V, the EF distribution was computed, and the EF magnitude at the position corresponding to the cell layer was extracted. This in vitro condition produced an EF range of 7.50‐8.00 mV mm^−1^ at the cell layer, which was then used as the target EF for translation to the in vivo setting (Figure S6).

Based on this target EF, an in vivo FEA model was then established based on the geometry of the ITO thin‐film‐based ES device attached to a mouse dorsal full‐thickness wound. The model included the ITO electrode, a commercial conductive hydrogel layer containing electrolytes within the hydrogel matrix to ensure conformal electrical contact, and the layered structure of mouse skin tissue and underlying tissue layers, including epidermis, dermis, fat, and muscle [35]. The electrical properties of each layer were assigned using reported conductivity values [36, 37], enabling estimation of EF distribution across the device‐hydrogel‐skin interface (Figure S7a,b).

The applied voltage in the in vivo model was then varied to identify a condition that could reproduce the target EF (7.50–8.00 mV mm^−1^) at the wound boundary. Among the tested conditions, an applied voltage of 5.00 V generated an EF distribution that matched the target range defined from the 0.50 V in vitro model. In the cross‐sectional FEA map, the EF propagated from the ITO thin‐film‐based ES device through the conductive hydrogel layer and into the underlying wounded skin tissue boundary (Figure 3b). Quantitative profiling across the cross‐section showed that the EF in the epidermal and dermal regions at the wound boundary (sky‐blue dashed line region in Figure 3b) was approximately 7.00–7.50 mV mm^−1^ (Figure 3c), which closely matched the 7.50–8.00 mV mm^−1^ EF experienced by cells under the in vitro condition.

To further characterize the EF distribution within each tissue layer, in‐plane EF maps were extracted from the epidermis and dermis, as well as from deeper fat and muscle layers. These maps showed that the EF was concentrated around the wound boundary, with stronger EF localization in the epidermal and dermal layers than in deeper tissues (Figure 3d and Figure S7c,d). Lateral EF profiles further confirmed that the EF reached its highest values at the wound boundary, particularly within the epidermis and dermis (Figure 3e). These results indicate that 5.00 V ES delivered through the skin‐conformal ES device can reproduce the effective EF (approximately 7.50 mV mm^−1^) within epidermal and dermal regions at the wound boundary.

To experimentally assess whether the FEA‐calculated electrical environment was reproduced under actual tissue conditions, we directly measured the local voltage profile using an oscilloscope. An external voltage of 5.00 V was applied to the skin‐device interface using the same power supply configuration employed for the in vivo experiments, while a fine‐tip probe was positioned at defined locations around the wound boundary to measure the local electrical potential. The potential differences measured over a defined distance were then used to estimate the local EF (Figure S8a).

The experimentally measured EFs at the wound boundary were approximately 9 mV mm^−1^ in both the in vivo and ex vivo conditions (Figure S8b). The measured values were slightly higher than the FEA prediction but showed a similar wound‐boundary‐localized distribution and remained within a comparable low‐intensity range. The biological relevance of this range is supported by previous studies showing that wound‐associated cells can respond to EFs as low as approximately 5 mV mm^−1^ [38]. Consistent with these reports, our previous study using the same ITO‐based ES platform demonstrated measurable responses in fibroblast and endothelial models at an EF of approximately 5 mV mm^−1^ [26]. Thus, despite being lower than the peak endogenous EFs reported at wound boundaries [39], the measured EF represents a low‐intensity electrical environment capable of eliciting cellular responses.

We next established an intermittent stimulation regimen that could provide sufficient biological stimulation while minimizing potential thermal effects. Although the 0.50 V condition used in vitro produced negligible temperature changes even during 24 h stimulation (Figure S9), the higher 5.00 V condition required for the in vivo configuration resulted in progressive heating during continuous operation. Continuous stimulation at 5.00 V produced a temperature increase of approximately 4°C after 5 h (Figure S10), a magnitude reported to influence wound repair through thermal effects [40]. We therefore adopted an intermittent stimulation strategy to minimize this potential thermal contribution.

Under the 2 h ES/1 h rest regimen, the temperature increase at the device‐contact region remained below 1.5°C relative to untreated skin (Figure S10). To determine whether the stimulation period could be further shortened while retaining the biological effect, we additionally evaluated a 1 h ES/1 h rest regimen for 7 days. Although this shorter regimen effectively limited thermal accumulation, it did not significantly enhance wound healing (Figure S11) or Ca_V_1.3 expression (Figure S12) compared with the corresponding control groups. These results indicate that a 1 h stimulation period was insufficient to induce a significant biological response, while prolonged continuous stimulation could introduce a thermal contribution. We therefore selected the 2 h ES/1 h rest regimen to elicit biological responses while limiting temperature elevation.

We then evaluated whether the selected regimen maintained a stable electrical and electrochemical environment during prolonged operation. During the 24 h stimulation period, we monitored the delivered current together with local pH and H_2_O_2_ generation (Figure 3f). The current remained stable, with a change of only approximately 3% between the beginning and end of the measurement period (Figure S13). Interfacial pH measured using the ratiometric fluorescent photoacid dye 6,8‐dihydroxypyrene‐1,3‐disulfonic acid disodium salt (DHPDS) [41, 42], changed by approximately 0.08 pH units over 24 h (Figure S14). H_2_O_2_ measured using an Amplex Red/horseradish peroxidase (HRP) assay remained at trace levels throughout stimulation, reaching a maximum concentration of only 0.05 µm over 24 h (Figure 3g and Figure S15) [43]. These results indicate that repeated cyclic stimulation maintained stable electrical delivery with minimal changes in the local electrochemical environment.

Based on these results, we proceeded with the 7‐day in vivo wound‐healing study using the established 5.00 V, 2 h ES/1 h rest regimen. Circular wounds (6 mm in diameter) extending to the dermal layer were created on the dorsal skin, and the ITO thin‐film‐based ES device was conformally attached to the wound through a conductive hydrogel interface and replaced every 24 h. Animals were divided into four groups: sham, device only, ES, and ES + Nifedipine. The sham group received neither device attachment nor ES, whereas the device only group received device attachment without ES. Both ES groups received the same stimulation regimen, with the ES + Nifedipine group additionally receiving 50 µM nifedipine applied directly to the wound once daily immediately before device attachment.

We first examined whether ES induced adaptive VGCC upregulation in the epidermal and dermal regions near the wound boundary. Wound tissues were collected on day 7 and analyzed for Ca_V_1.3 expression signal by immunohistochemistry (IHC). Within the wound region, normalized Ca_V_1.3 fluorescence intensity was significantly higher in the ES‐treated group, showing a 37.1 ± 8.40% increase relative to the sham groups. The device only group showed Ca_V_1.3 expression levels comparable to those of the sham group, indicating that the device itself, including the conductive hydrogel layer, does not contribute to VGCC upregulation. Notably, Ca_V_1.3 expression in the ES + Nifedipine group remained comparable to that in the sham group. By suppressing VGCC‐mediated Ca^2+^ influx, nifedipine prevented the adaptive increase in Ca_V_1.3 expression induced by ES (Figure 3h,i, and Figure S16). These findings indicate that ES delivered under EF‐matched conditions induces VGCC upregulation in wound tissue, consistent with the in vitro observations (Figure 2d,e).

To further define the cellular context of the Ca_V_1.3 response in vivo, we examined its spatial association with α‐smooth muscle actin (α‐SMA), a marker associated with activated fibroblast populations during wound repair. The Ca_V_1.3/α‐SMA‐positive area was 44.94 ± 5.96% higher in the ES‐treated group than in the sham group (Figure S17), indicating increased Ca_V_1.3 expression within α‐SMA‐positive stromal regions. This tissue‐level observation is consistent with the increased Ca_V_1.3 expression observed in NIH3T3 fibroblasts following ES in vitro (Figure S4) and further supports the presence of the Ca_V_1.3 response in wound‐relevant stromal contexts.

We next examined whether ES‐induced VGCC expression is associated with enhanced wound repair. Serial macroscopic imaging of wound regions over 7 days showed accelerated wound closure in the ES‐treated group compared with sham, device only, and ES + Nifedipine groups (Figure 3j). Quantitative analysis of wound area normalized to day 0 confirmed that the ES‐treated group exhibited faster closure kinetics, with 63.2 ± 11.7% greater wound closure relative to sham groups at day 7. In contrast, the ES + Nifedipine group lost the ES‐associated enhancement in wound closure, with wound closure reduced to levels comparable to those of the sham and device only groups (Figure 3k). Together, the suppression of both ES‐induced Ca_V_1.3 upregulation and the associated wound‐healing response by nifedipine supports a functional contribution of VGCC‐mediated Ca^2+^ signaling to enhanced wound repair under ES.

Because no anti‐contraction measures were used in our wound model, macroscopic wound closure may reflect both contraction and re‐epithelialization and was therefore interpreted together with histological evidence of tissue repair. Hematoxylin and eosin (H&E) staining of tissues collected on day 7 revealed reduced wound length in the ES group compared with the sham, device only, and ES + Nifedipine groups, indicating advanced re‐epithelialization and tissue reconstruction (Figure S18). In addition, Masson's trichrome (MT) staining showed increased collagen deposition within the wound region in the ES‐treated group, reflecting enhanced extracellular matrix formation associated with wound healing (Figure 3l,m). These findings collectively demonstrate that ES, when applied under EF‐matched conditions, increases VGCC expression in wound tissue and enhances wound tissue repair, as supported by histological evidence of re‐epithelialization and extracellular matrix deposition in addition to macroscopic wound closure.

### 2.3 ES‐Induced Vascular Remodeling and Macrophage‐Associated Marker Changes in Wound Tissue

To further understand whether ES‐induced VGCC upregulation is associated with broader regenerative remodeling during wound healing, we also examined tissue‐level processes beyond overall wound closure and collagen deposition. While the preceding analyses demonstrated that ES promotes Ca_V_1.3 upregulation together with accelerated wound healing, Ca_V_1.3‐mediated Ca^2+^ signaling is also known to regulate multiple cellular programs involved in tissue regeneration [44, 45], including angiogenic activity [46] and inflammatory regulation [47, 48]. Because these coordinated biological responses critically determine the quality and progression of wound repair [49, 50], we investigated whether ES‐induced enhancement of VGCC activity is accompanied by vascular‐associated remodeling and macrophage‐associated inflammatory regulation within the wound microenvironment (Figure 4a).

**FIGURE 4:**
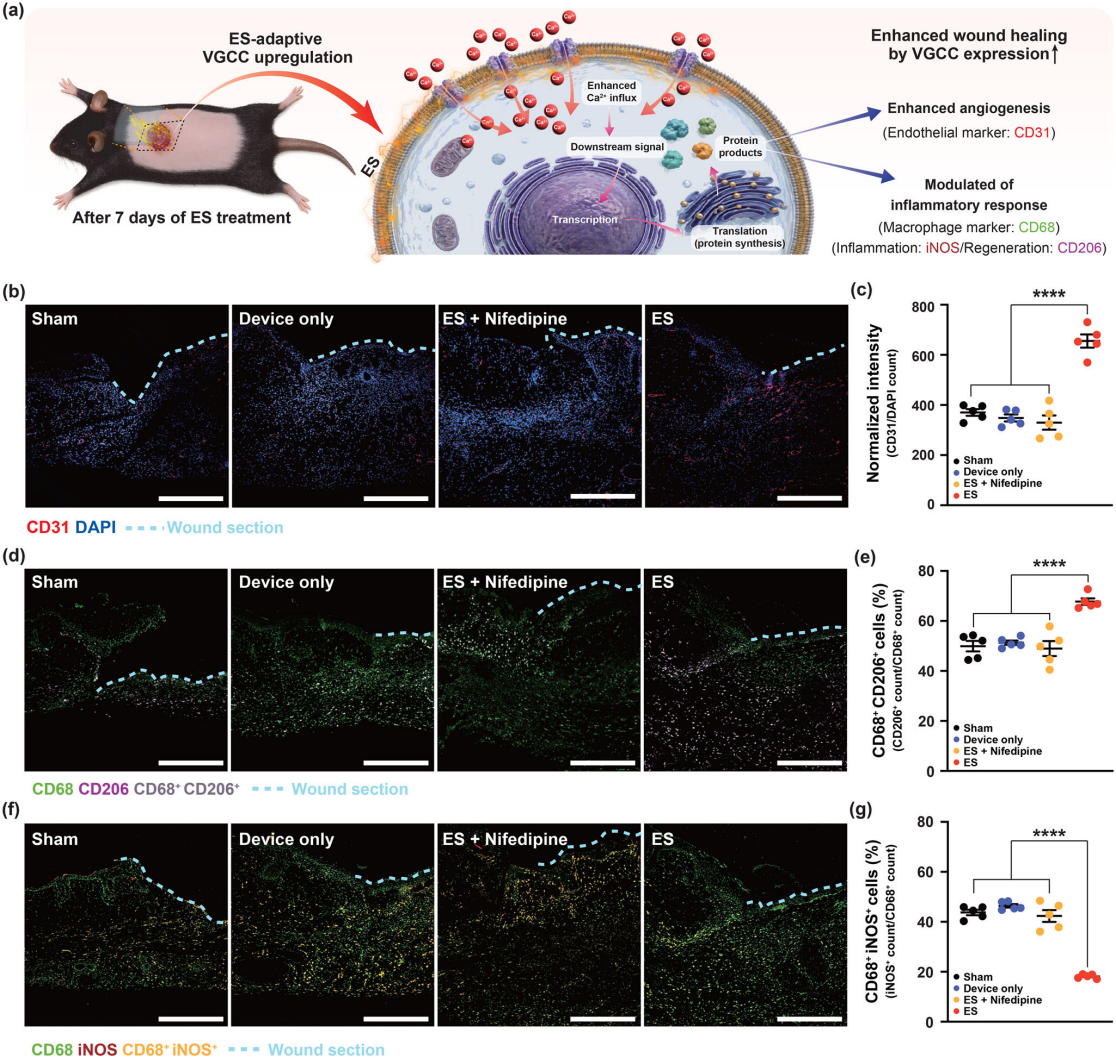
Angiogenic and inflammatory regulation associated with VGCC upregulation. (a) Schematic illustration of angiogenic and macrophage‐associated responses within the wound microenvironment associated with ES‐induced VGCC upregulation. (b) Representative immunofluorescence images of wound tissue sections collected on day 7 from sham, device only, ES + Nifedipine, and ES‐treated groups stained for CD31 (red) and DAPI (blue). The light‐blue dashed curved line indicates the wound region (scale bar =  400 µm, all single‐channel immunofluorescence images are displayed in Figure S16). (c) Quantification of normalized CD31 intensity relative to DAPI count (*n*  =  5 per group, all *p*‐values are listed in Table S7). (d) Representative immunofluorescence images of day 7 wound sections showing CD68 (green), CD206 (magenta), and CD68^+^CD206^+^ cells (gray) (scale bar =  400 µm, all single‐channel and merged‐channel images are displayed in Figure S21). (e) Quantification of the percentage of CD206^+^ cells within the CD68^+^ population in each group (*n*  =  5 per group, all *p*‐values are listed in Table S8). (f) Representative day 7 wound section immunofluorescence images displaying CD68 (green), iNOS (red), and CD68^+^iNOS^+^ cells (yellow) (scale bar =  400 µm, all single‐channel and merged‐channel images are displayed in Figure S23). (g) Quantification of the ratio of iNOS^+^ cells within the CD68^+^ population (*n*  =  5 per group, all *p*‐values are listed in Table S9). Data in Figure 4c,e,g are presented as mean ± s.e.m., and statistical analyses were performed using one‐way ANOVA followed by Tukey's multiple‐comparison test. Statistical significance is indicated as ****p < 0.0001.

We first evaluated vascular remodeling by staining day 7 wound tissues for CD31, an endothelial cell marker commonly used to assess vascular structures during tissue repair [51, 52]. IHC staining revealed a stronger CD31 signal in the ES‐treated group compared with sham, device only, and ES + Nifedipine groups (Figure 4b and Figure S16). Quantitative analysis of normalized CD31 intensity (CD31/DAPI count) showed a 76.9 ± 9.50% increase in the ES group relative to the sham group, whereas this increase was markedly attenuated in the ES + Nifedipine group (Figure 4c). Consistent with these findings, histological analysis revealed approximately 2.5‐fold higher blood vessel counts in the ES group than in the sham group, whereas this increase was not observed in the ES + Nifedipine group (Figure S19).

To further define the relationship between the Ca_V_1.3 response and vascular remodeling in vivo, we analyzed the spatial co‐localization of Ca_V_1.3 with CD31. The Ca_V_1.3/CD31‐positive area increased by 47.7 ± 5.51% in the ES‐treated group relative to the sham group (Figure S20), indicating increased Ca_V_1.3 expression within CD31‐positive vascular regions. This tissue‐level observation is consistent with the Ca_V_1.3 response observed in HUVECs in vitro (Figure S3) and supports the presence of the ES‐associated Ca_V_1.3 response in vascular regions of the wound tissue. Collectively, these findings indicate that ES promotes vascular remodeling within the wound tissue, consistent with the pro‐regenerative effects associated with enhanced VGCC‐mediated Ca^2+^ signaling following ES, and suggest a contribution of L‐type VGCC‐mediated Ca^2+^ signaling to this response. The increased CD31‐positive endothelial signal and higher number of histologically identified vascular structures provide structural evidence of increased vascular density and remodeling. While functional blood perfusion was not directly assessed, these findings are consistent with the broader regenerative effects of bioelectric stimulation, including the modulation of Ca^2+^‐mediated cellular responses and the local wound microenvironment [53, 54].

Because successful wound repair requires coordinated inflammatory regulation alongside vascular remodeling [55, 56], we then examined macrophage‐associated responses within the wound microenvironment. Wound tissue sections collected on day 7 were stained for CD68, a widely used macrophage‐associated marker that labels cells of the monocyte/macrophage lineage [57], to estimate overall macrophage abundance in tissue sections. No significant difference in normalized CD68 intensity (CD68/DAPI count) was observed among sham, device only, ES + Nifedipine, and ES groups (Figure 4d, Figures S21 and S22), indicating that ES did not substantially alter overall macrophage‐associated abundance in the wound tissue.

Although the overall macrophage‐associated abundance remained comparable across groups, macrophages can adopt distinct functional states during wound healing, including inflammatory and wound healing‐associated states [58, 59]. To characterize changes in macrophage‐associated marker profiles, CD206 and iNOS were examined as markers associated with wound repair and inflammatory responses, respectively. Notably, in the ES‐treated group, the proportion of CD206‐positive cells among CD68‐positive cells (CD68^+^CD206^+^) was 35.7 ± 6.34% higher than in the sham group (Figure 4e and Figure S21). This increase was not observed in the ES + Nifedipine group, in which the CD68^+^CD206^+^ proportion remained comparable to those of the sham and device only groups.

Similarly, the proportion of iNOS‐positive cells among CD68‐positive cells (CD68^+^iNOS^+^) decreased by 58.4 ± 1.35% following ES, whereas the ES + Nifedipine group showed iNOS‐associated expression comparable to the sham and device only groups (Figure 4f,g, and Figure S23). Consequently, the CD206^+^/iNOS^+^ ratio was approximately 3.3‐fold higher in the ES group relative to the sham group (Figure S24). These findings indicate that ES alters the balance of CD206 and iNOS marker expression within wound‐associated macrophages.

Taken together, we found that ES in our system not only induces Ca_V_1.3 upregulation but also promotes vascular‐associated remodeling and alters the CD206/iNOS macrophage‐associated marker balance within the wound tissue. Notably, these tissue‐level responses were attenuated by nifedipine, with the corresponding measurements remaining comparable to those of the sham and device only groups, supporting a contribution of L‐type VGCC‐mediated Ca^2+^ signaling to the regenerative effects of ES. These tissue‐level responses may be partially associated with the upregulation of *PI3K‐AKT‐mTOR* and *MEK‐ERK* signaling pathways following ES treatment observed in vitro (Figure 2a,b), as these pathways are closely involved in angiogenesis and inflammatory regulation in wound tissue [60, 61]. Although the detailed mechanisms linking VGCC upregulation to these tissue‐level regenerative responses require further investigation, these findings suggest that the adaptive cellular responsiveness induced by ES extends to coordinated regenerative remodeling within the wound microenvironment.

### 2.4 Transcriptional and Proteomic Analyses of Human Skin Cell Adaptation to ES

Having established adaptive cellular responses to ES in both in vitro and in vivo murine models, we also investigated the translational relevance of these findings using human epidermal melanocytes (HEMa) as a representative human skin cell model (Figure 5a). Similar to the B16F10 cell experiments, HEMa cells were cultured on an ITO thin‐film electrode and exposed to ES over an applied voltage range of 0.00–0.75 V for 48 h (Figure S25). Among the tested voltages, 0.50 V elicited the most enhanced proliferative behavior and was therefore selected for subsequent HEMa cell ES experiments.

**FIGURE 5:**
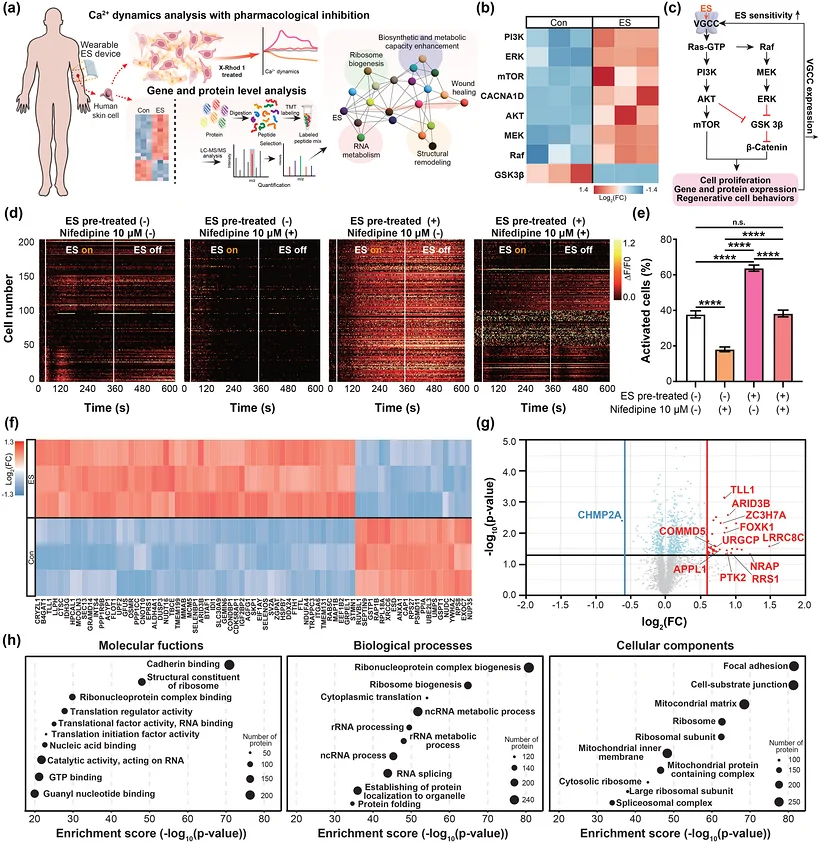
Transcriptional and proteomic remodeling in human skin cells upon ES. (a) Schematic workflow of transcriptional and proteomic analyses using HEMa cells cultured under non‐stimulated control (Con) or ES conditions (ES). (b) Heatmap showing relative expression levels of targeted genes exhibiting statistically significant differences between the Con and ES‐treated groups, as analyzed by t‐test. Red and blue indicate upregulation and downregulation, respectively, relative to the Con group (*n*  =  3 per group, all *p*‐values are listed in Table S10). (c) Schematic illustration of the proposed ES‐adaptive signaling pathways in skin cells. (d) Heatmaps depicting X‐Rhod‐1 AM fluorescence responses (ΔF/F_0_) from 200 individual HEMa cells randomly selected from at least four independent experiments throughout the Ca^2+^ imaging period. Cells in the ES pre‐treated group were exposed to 0.50 V ES for 48 h before imaging, whereas the nifedipine group received 10 µM nifedipine. During imaging, ES was delivered from 30 to 330 s, as indicated by the white lines. (e) Quantification of the fraction of cells classified as activated during the subsequent ES period. Prior ES exposure significantly increased the proportion of activated cells, whereas nifedipine treatment reduced this response. For each condition, 50 cells were randomly selected from each of four independent biological experiments (200 cells in total), and the percentage of activated cells was calculated separately for each experiment. Statistical analysis was performed using these independent replicate‐level measurements (n = 4 per condition) by one‐way ANOVA followed by Tukey's multiple‐comparison test, with individual *p*‐values provided in Table S11. Statistical significance is indicated as ****p < 0.0001. (f) Heatmap of 75 significantly regulated proteins identified from approximately 3,000 detected proteins in Con‐ and ES‐treated groups (*n*  =  3 independent biological replicates, all *p*‐values are provided in Table S12). (g) Volcano plot showing proteins with statistically significant differences in abundance between the Con‐ and ES‐treated groups (*n*  =  3 independent biological replicates). Significantly upregulated proteins are shown in red, downregulated proteins in blue, and non‐significant proteins in gray. (h) GO enrichment analysis of significantly regulated proteins, categorized into molecular functions, biological processes, and cellular components. Dot size represents the number of proteins enriched in each GO term, and the x‐axis shows the enrichment score (‐log_10_(*p*‐value)).

We first investigated whether the ES‐adaptive transcriptional changes identified in B16F10 cells could also be reproduced in HEMa cells. The targeted genes comprised VGCC‐associated genes and Ca^2+^‐dependent signaling nodes, including components of the *PI3K*‐*AKT*‐*mTOR* and *Raf*‐*MEK*‐*ERK* pathways, all of which showed notable upregulation in the B16F10 screening (Figure 2a,b). We also included *GSK3β*, which exhibited reduced expression in B16F10 cells following ES treatment (Figure 2a,b). Similar to our findings in B16F10 cells, ES at 0.50 V for 48 h increased the expression of VGCC‐ and Ca^2+^‐downstream signaling‐related genes in HEMa cells, whereas *GSK3β* expression was significantly reduced relative to non‐stimulated controls (Figure 5b).

Based on these transcriptional changes, the adaptive responses of HEMa cells to ES were schematically summarized (Figure 5c). In this scheme, ES‐induced VGCC upregulation is associated with increased Ca^2+^ influx, which subsequently activates downstream *PI3K*‐*AKT*‐*mTOR* and *Raf*‐*MEK*‐*ERK* signaling pathways. Concurrently, *GSK3β* expression was suppressed, likely reflecting inhibitory regulation through *AKT*‐dependent phosphorylation or ERK‐associated downstream signaling [62]. Because these pathways are closely involved in biosynthetic activity, metabolism, and protein synthesis, their activation may facilitate sustained expression of VGCC‐associated signaling components during sustained ES exposure and reinforce adaptive cellular responses. Together, these findings suggest that ES promotes a VGCC‐associated transcriptional program in HEMa cells and strengthens downstream signaling networks associated with proliferative, biosynthetic, and wound repair‐related cellular responses.

We next examined whether these molecular changes were accompanied by altered Ca^2+^ responsiveness. Because of the low transfection efficiency of GCaMP6s in HEMa cells, intracellular Ca^2+^ dynamics were monitored using the cell‐permeable indicator X‐Rhod‐1 AM after 48 h of ES [63]. Upon subsequent stimulation, HEMa cells with prior ES exposure showed a greater Ca^2^^+^ response than cells without prior exposure (Figure 5d). Cells exhibiting an increase in X‐Rhod‐1 AM fluorescence (ΔF/F_0_) of more than 10% were classified as responsive, and the fraction of responsive cells was 68.9 ± 10.0% higher in the ES pre‐treated group (Figure 5e). Importantly, nifedipine attenuated this enhanced Ca^2+^ response, indicating that L‐type VGCC activity contributes to the increased Ca^2+^ responsiveness induced by sustained ES (Figure 5d). These results demonstrate that sustained ES enhances subsequent Ca^2+^ responsiveness in HEMa cells, providing a functional correlate of the VGCC‐associated molecular changes.

We further examined the contribution of VGCC‐mediated Ca^2+^ signaling to the cellular response to ES. Nifedipine significantly attenuated ES‐induced HEMa cell proliferation compared with ES alone (Figure S26), supporting the functional involvement of L‐type VGCC‐mediated Ca^2+^ influx in the proliferative response. Together, the transcriptional, Ca^2+^ imaging, and pharmacological results indicate that HEMa cells adapt to ES through enhanced VGCC‐mediated Ca^2+^ signaling.

Having established that the ES‐induced transcriptional changes were accompanied by enhanced functional Ca^2+^ responsiveness, we next investigated whether the broader adaptive cellular state was also reflected at the proteomic level. To this end, tandem mass tag (TMT)‐based quantitative proteomics was performed using control and ES‐treated HEMa samples. Principal component analysis (PCA) showed clear separation between control and ES‐treated groups, with PC1 accounting for 65.4% of the total variance (Figure S27), indicating that ES induced a distinct proteomic state in HEMa cells.

TMT‐based quantitative proteomic analysis detected approximately 3,000 proteins across control and ES‐treated HEMa samples. Among these, 75 proteins exhibiting significant abundance changes were visualized by heatmap analysis, which clearly distinguished ES‐treated cells from controls (Figure 5f). Volcano plot analysis further identified 38 upregulated proteins and 1 downregulated protein following ES exposure. Notably, several prominently upregulated proteins were functionally associated with ion transport, cytoskeletal organization, mitochondrial function, and protein modification, including LRRC8C (log_2_FC ≈ 2.79), NRAP (2.30), COA3 (2.13), and TMT1A (2.03) (Figure 5g). The upregulation of these proteins suggests that ES promotes coordinated remodeling of protein programs associated with cellular signaling responsiveness and repair‐associated cellular behavior.

To further define the biological significance of these proteomic alterations, functional annotation analysis was performed. ES‐regulated proteins were associated with several major biological categories, including signaling integration (PTK2, APPL1, HIPK2) [64], transcriptional and post‐transcriptional regulation (ARID3B, FOXK1, ZC3H7A) [65], ribosome biogenesis (RRS1, GTF2F2) [66], mitochondrial metabolism (COA3, TIMM21, SURF1, NFU1) [67], and structural remodeling (NRAP, TLL1, SYNE1) [68], along with ion‐transport regulators such as LRRC8C and LRRC8A (Figure S28). These results suggest that ES promotes broad proteomic remodeling across biosynthetic, metabolic, structural, and ion transport‐associated cellular programs.

Consistent with these findings, gene ontology (GO) enrichment analysis of the upregulated candidate ES‐responsive proteins identified ribosome biogenesis, RNA metabolism, translation, mitochondrial activity, cytoskeletal organization, and focal adhesion as significantly enriched processes after Benjamini‐Hochberg correction (adjusted *p* < 0.05) (Figure 5h). Network‐based analysis further revealed interconnected protein modules associated with signaling integration, RNA processing, energy production, and cytoskeletal remodeling (Figure S29). Together, these pathway‐level analyses suggest that the ES‐associated proteomic changes are linked to coordinated biosynthetic, metabolic, and structural remodeling that may support the enhanced VGCC expression and proliferative behavior observed following stimulation.

Overall, these data demonstrate that ES induces VGCC‐associated transcriptional changes in HEMa cells and is accompanied by broader proteomic remodeling related to biosynthesis, metabolism, and cell‐structural remodeling. These coordinated transcriptional and proteomic adaptations may facilitate sustained VGCC upregulation and enhanced proliferative, wound healing‐associated cellular behavior. These findings extend the observation from murine in vitro and in vivo models to a human cellular context and suggest that ES‐induced VGCC‐associated sensitization could be conserved across species at the skin‐electrode interface.

## 3 Conclusions

In this work, we established that sustained ES dynamically reshapes the functional state of skin cells at the bioelectronic interface by enhancing VGCC‐associated Ca^2+^ responses. Using a transparent ITO‐based bioelectronic platform together with integrated transcriptional, proteomic, immunological, and live‐cell Ca^2+^ imaging analyses, we identified VGCC‐associated Ca^2+^ sensitization as a key mediator of this adaptive ES‐responsive state in both mouse and human skin‐derived cell models in vitro. We further extended these findings to mouse wound tissue under experimentally validated ES conditions established through FEA‐guided stimulation, direct EF measurements, and electrical and electrochemical stability assessments. ES increased Ca_V_1.3 expression across wound tissue, with increased Ca_V_1.3 signals observed in stromal and vascular regions, and promoted regenerative responses characterized by accelerated wound closure, collagen deposition, vascular remodeling, and changes in macrophage‐associated marker profiles. Importantly, pharmacological inhibition of L‐type VGCCs with nifedipine attenuated these ES‐induced effects, further underscoring the functional contribution of VGCC‐mediated Ca^2+^ signaling to ES‐assisted tissue repair. Although the detailed feedback mechanisms underlying sustained VGCC regulation remain to be fully elucidated, this study provides insight into how skin cells adapt to sustained electrical stimuli at the bioelectronic interface. Collectively, these findings highlight the importance of investigating adaptive cellular responsiveness during ES‐assisted wound healing and suggest a framework for developing adaptive bioelectronic therapies centered on VGCC‐associated signaling.

## 4 Methods

### 4.1 Chemicals and Reagents

ITO thin‐film sheets (L × W × thickness, 30.48 cm × 30.48 cm × 0.0127 cm, surface resistivity, 60 Ω sq^−1^) were purchased from Sigma‐Aldrich (USA). B16F10 cells and HUVEC were obtained from ATCC (USA). NIH3T3 cells were obtained from Korean Cell Line Bank (Korea), and HEMa cells were obtained from PromoCell (Germany). B16F10 cells were cultured in high‐glucose Dulbecco's modified Eagle medium (DMEM, WelGene, South Korea), whereas HEMa cells were maintained in melanocyte growth medium supplemented with SupplementMix (PromoCell, Germany). Phosphate‐buffered saline (PBS), Dulbecco's phosphate‐buffered saline (DPBS), distilled water (DW), and GlutaMAX were purchased from Gibco (South Korea). Characterized fetal bovine serum (FBS) was purchased from HyClone (Cytiva, Ireland). High‐performance liquid chromatography (HPLC)‐grade water was obtained from Merck (Germany). HPLC‐grade methanol and acetonitrile (> 99%) were purchased from J.T. Baker (Radnor, PA, USA). Sodium chloride (NaCl, ≥99%), potassium chloride (KCl, ≥99%), magnesium chloride hexahydrate (MgCl_2_·6H_2_O, ≥99%), calcium chloride dihydrate (CaCl_2_·2H_2_O, ≥99%), HEPES (H3375, ≥99.5%), and D‐glucose (≥99.5%) were obtained from Sigma‐Aldrich (USA). Sodium hydroxide solution (NaOH, 1 N) was purchased from Duksan. Formic acid was obtained from Sigma‐Aldrich (USA). All other chemicals were analytical grade.

### 4.2 Cell Culture and In Vivo Platform Preparation

The ITO‐PET film measuring 120 mm × 25 mm × 0.0127 cm was cut into dimensions for a 1‐well cell culture slide platform, whereas a film measuring 10 mm × 20 mm × 0.0127 cm was produced for the in vivo device. The ITO thin‐film was designed using AutoCAD software (Autodesk, USA) and cut using an ultraviolet (UV) laser (basiCube 10, SCANLAB, Germany) at 355 nm. The cutting procedure consisted of six iterative cycles under the following defined parameters: laser velocity of 50 mm s^−1^, 70% power, a frequency of 1000 kHz, a laser on‐time cycle of 300 µs, and a laser off‐time cycle of 100 µs. After cutting, the ITO thin‐film was cleaned with a tissue soaked in 70% ethanol to remove residual particulate matter generated during laser cutting. The film was then placed under a clean bench air blower with UV illumination for 60 min. Finally, the films were used to fabricate the cell culture platform and the in vivo device.

### 4.3 In Vitro ES Protocol

ES was applied to cells cultured on the ITO thin‐film electrode using a direct current power supply (Agilent E3641A, Agilent, USA). The ITO thin‐film was connected to the power supply using copper wires and alligator clips to maintain stable electrical contact throughout stimulation. For all in vitro experiments, cells were seeded onto the ITO thin‐film electrode at a density of 80,000 cells per well and allowed to attach before stimulation. Defined voltages ranging from 0.00 to 1.00 V were then applied across the ITO thin‐film electrode to induce ES. All in vitro stimulation experiments were performed inside a standard cell culture incubator maintained at 37°C, 95% relative humidity, and 5% CO_2_ to preserve physiological culture conditions during stimulation. The duration of ES was adjusted according to cell‐type‐specific growth characteristics. B16F10 and NIH3T3 cells, with reported doubling times of approximately 17 h and 20–21 h [69, 70], respectively, were stimulated for 24 h. In contrast, HUVEC and HEMa cells, which exhibit distinct growth characteristics and more limited proliferative capacity [71, 72], were stimulated for 48 h to allow sufficient time for cellular responses. Unless otherwise stated, these ES durations were used for the respective in vitro experiments. B16F10 cells (0.50 V) and NIH3T3 (0.50 V), which have a relatively short doubling time, were stimulated for 24 h, whereas HEMa cells (0.50 V) and HUVEC (0.25 V), which proliferate more slowly, were stimulated for 48 h. Unless otherwise stated, these ES durations were used for the corresponding in vitro experiments.

### 4.4 Cell Proliferation and Viability Assay

Cell proliferation was evaluated using a Cell Counting Kit‐8 (CCK‐8, Dojindo Laboratories, Japan) in B16F10 and HEMa cells. After completion of ES for 24 h (B16F10) or 48 h (HEMa), the culture medium was carefully removed from each sample and replaced with fresh complete growth medium containing CCK‐8 reagent at a final concentration of 10% (v/v). Samples were then incubated for 90 min at 37°C in a humidified incubator with 5% CO_2_, under light‐protected conditions to prevent reagent degradation. Following incubation, the reacted medium was transferred to a 96‐well plate, and absorbance was measured at 450 nm using a microplate reader (Spark, Switzerland). The measured absorbance was used as an index of the number of metabolically active cells. Each experimental condition was analyzed in triplicate unless otherwise stated.

For nifedipine treatment, nifedipine (Sigma‐Aldrich, USA) was dissolved in dimethyl sulfoxide (DMSO, Sigma‐Aldrich, USA) to prepare a stock solution and diluted in DMEM to a final concentration of 10 µm (4 mL per 1‐well culture slide) before use. B16F10 cells were cultured under standard culture conditions with this solution for 24 h. HEMa cells were similarly treated with 10 µM nifedipine prepared in melanocyte growth medium for 48 h.

### 4.5 Live and Dead Assays

Cell viability was assessed by calcein acetoxymethyl ester (calcein AM) and ethidium homodimer‐I (EthD‐I) staining in B16F10 and HEMa cells. After ES treatment for 24 h for B16F10 cells or 48 h for HEMa cells, the culture medium was aspirated, and the samples were gently washed with PBS. Cells were then incubated with 100 µL of culture medium containing calcein AM 0.05 µL and EthD‐I 0.2 µL (Biotium, USA) for 30 min at room temperature in the dark. Fluorescence images were acquired using a fluorescence microscope (ZEISS Axio Imager 2, ZEISS, Germany) under identical imaging settings for all groups. Viable cells were identified by green calcein fluorescence, whereas dead cells were identified by red EthD‐I fluorescence.

### 4.6 Gene Expression Analysis

Quantitative real‐time polymerase chain reaction (RT‐qPCR) analysis of 69 genes for B16F10 and 8 genes for HEMa was performed on cells cultured on a 1‐well culture slide, with or without 0.50 V ES. Total RNA was extracted using the RNeasy Protect Cell Kit (Qiagen, USA) according to the manufacturer's instructions. Briefly, cells were lysed in lysis buffer, and genomic DNA was removed. Ethanol was added to the lysate for further washing steps using a manufacturer‐provided purification spin column. Finally, total RNA was collected using a spin column and eluted with RNase‐free water. The quality (A260/A280 ratio) and concentration of RNA was assessed using a NanoDrop UV‐visible spectrophotometer (Thermo Fisher Scientific, USA). The RNA was reverse‐transcribed into cDNA using a reverse transcription kit (Takara Korea Biomedical Inc., South Korea). RT‐qPCR was performed using a commercial SYBR Supermix Kit. The RT‐qPCR amplification was carried out under the following conditions: an initial hold at 50°C for 2 min, followed by denaturation at 95°C for 10 s, annealing at 60°C for 15 s, and extension at 72°C for 30 s. The housekeeping gene glyceraldehyde‐3‐phosphate dehydrogenase (GAPDH) was used as an endogenous control. Relative gene expression levels were calculated using the 2^(‐ΔΔCt)^ method. Each experimental condition was performed in triplicate. Differential gene expression was analyzed using the one‐factor statistical analysis module in MetaboAnalyst 6.0. Volcano plots were generated using a fold‐change threshold of 1.5, a significance threshold of *p* < 0.05. Multiple testing was corrected using a two‐stage BKY procedure with an FDR threshold of 10%. Primer sequences for the forward (Fp) and reverse (Rp) primers are listed in Tables S13 and S14.

### 4.7 ICC Staining

Cells cultured on ITO thin‐film electrodes mounted on 1‐well slides, with or without ES treatment, were prepared for ICC. After removal of the culture medium, samples were washed three times with 1 mL PBS and fixed with 500 µL of 4% paraformaldehyde in PBS for 15 min at room temperature. Fixed cells were washed three times with PBS for 5 min each on a rocking platform, permeabilized with 500 µL of 0.2% 4‐(1,1,3,3‐tetramethylbutyl) phenyl‐polyethylene glycol (Triton X‐100, Sigma‐Aldrich, USA) in PBS for 10 min at room temperature, and washed three times again with PBS. Samples were blocked with 500 µL of 5% goat serum in PBS for 30 min at room temperature. The Ca_V_1.3 primary antibody was diluted 1:200 in antibody dilution buffer containing 1% bovine serum albumin in PBS and added to each well at 500 µL per sample. Cells were incubated with the primary antibody overnight at 4°C, then washed three times with PBS for 5 min each. Fluorescent secondary antibodies were diluted 1:1000 in the same antibody dilution buffer and incubated with the samples for 30 min at room temperature in the dark. After secondary antibody incubation, samples were washed three additional times with PBS. The stained ITO thin‐films were carefully detached using forceps, briefly blotted to remove excess liquid, and mounted onto microscope slides with VECTASHIELD HardSet Antifade Mounting Medium with DAPI (H‐1500‐10, Vector Laboratories, USA), with the cell side facing downward. Mounted samples were dried at room temperature and sealed before imaging. Fluorescence images were acquired using a fluorescence microscope with appropriate filter sets. Antibody information is provided in Table S15.

### 4.8 Western Blot

B16F10 cells were seeded at a density of 300 000 cells in a 1‐well culture slide (SPL, USA) with an ITO substrate attached to the bottom. On the following day, the ITO substrate was connected to a power supply, and cells were either stimulated at 0.50 V for 24 h or left unstimulated under the same culture conditions. After treatment, the cells were lysed in radioimmunoprecipitation assay (RIPA) Lysis and Extraction Buffer (89901, Thermo Fisher Scientific, USA) supplemented with protease/phosphatase inhibitor cocktail (P3300‐005, GenDEPOT, USA). The lysates were kept on ice, vortexed every 10 min for three cycles, and centrifuged at 14 000 x g for 30 min at 4°C. The supernatants were collected, and protein concentrations were determined using a bicinchoninic acid (BCA) assay kit (23227, Thermo Fisher Scientific, USA). Equal amounts of protein were separated on a 6% sodium dodecyl sulfate‐polyacrylamide gel electrophoresis (SDS‐PAGE) gel and transferred onto a polyvinylidene fluoride (PVDF) membrane using transfer buffer containing 10% methanol for 2 h. The membranes were blocked with 5% bovine serum albumin (BSA) for 30 min and incubated overnight at 4°C with rabbit anti‐Ca_V_1.3 primary antibody (diluted 1:1000) or mouse anti‐β‐actin antibody (diluted 1:10 000) in 5% BSA. After washing three times with PBS containing 0.05% Tween‐20 (PBST), the membranes were incubated separately with the corresponding HRP‐conjugated anti‐rabbit or anti‐mouse secondary antibody diluted 1:10 000 in 5% BSA for 1 h at room temperature. Following three additional washes with PBST, signals were developed using a chemiluminescent HRP substrate (34095, Thermo Fisher Scientific, USA), and images were acquired using an ImageQuant 800 imaging system. The antibodies used in this study are listed in Table S15.

### 4.9 Plasmid Transfection

B16F10 cells seeded on a 1‐well culture slide were transfected at 70–80% cell confluency after 24 h of culturing (Con and 0.50 V ES). Specifically, each well received 2 µg of plasmid DNA, 4 µL of P3000, and 6 µL of Lipofectamine 3000 reagent (L3000, Invitrogen, USA) in 200 µL Opti‐MEM^TM^ (Gibco, South Korea). pGP‐CMV‐GCaMP6s (Addgene plasmid no. 40753, USA) was used for transfection. pGP‐CMV‐GCaMP6s was a gift from Douglas Kim & GENIE Project (Addgene plasmid no. 40753, http://n2t.net/addgene:40753, RRID: Addgene_40753).

### 4.10 Calcium Imaging

B16F10 cells were cultured on 1‐well slide plates with or without 0.50 V ES for 24 h before calcium imaging. Immediately before imaging, the culture medium was replaced with 4 mL Tyrode's solution. GCaMP6s fluorescence was recorded using an inverted fluorescence microscope (in situ cell/material analyzer, NFEC‐2024‐12‐301386) at 1 frame s^−1^. After a 30 s baseline recording, 0.50 V ES was applied for 300 s to evaluate cellular responsiveness to electrical input [73]. Stimulation was then stopped, and fluorescence was recorded for an additional 270 s to monitor post‐stimulation Ca^2+^ dynamics. Single‐cell fluorescence traces were analyzed using ImageJ (version 1.54p, Wayne Rasband, National Institutes of Health, USA). Baseline fluorescence (F_0_) was calculated as the average fluorescence intensity during the initial 30 s before ES application. Calcium responses were expressed as ΔF/F_0_. For quantitative analysis, 50 B16F10 cells were randomly selected from each of three independent biological experiments, yielding a total of 150 cells per condition. The fraction of activated cells was calculated separately for each biological replicate, and the replicate‐level values were used as the statistical unit (*n* = 3 per condition). Individual‐cell ΔF/F_0_ traces were used for visualization of Ca^2+^ dynamics.

For calcium imaging in HEMa cells, cells were cultured with or without 0.50 V ES for 48 h before imaging. Because HEMa cells exhibited limited transfection efficiency with genetically encoded calcium indicators, intracellular Ca^2+^ dynamics were monitored using the cell‐permeable fluorescent calcium indicator X‐Rhod‐1 AM (Invitrogen, USA). HEMa cells were incubated with 5 µM X‐Rhod‐1 AM for 30 min at room temperature, followed by washing to remove extracellular dye. The cells were subsequently maintained in Tyrode's solution for live‐cell imaging. Following a 30 s baseline recording, 0.50 V ES was applied for 300 s, followed by an additional 270 s recording period after cessation of stimulation. X‐Rhod‐1 fluorescence traces were analyzed on a single‐cell basis using ImageJ. For HEMa cells, 50 cells were randomly selected from each of four independent biological experiments, yielding a total of 200 cells per condition. The fraction of responsive cells was calculated separately for each biological replicate, and the replicate‐level values were used for statistical analysis (*n* = 4 per condition).

For Ca^2+^ imaging of nifedipine‐treated cells, the culture medium was replaced 30 min before imaging with the corresponding growth medium containing 10 µM nifedipine. DMEM was used for B16F10 cells, whereas melanocyte growth medium was used for HEMa cells. For the corresponding vehicle controls, an equivalent volume of DMSO was added to each cell type‐specific growth medium.

### 4.11 FEA‐Based EF Profile Calculation

FEA was conducted using COMSOL Multiphysics (COMSOL Inc., Sweden) to analyze EF distributions in the in vitro and in vivo stimulation configurations [74]. Three‐dimensional geometries were constructed and solved using the Electric Currents interface under steady‐state conditions. The analysis was performed to calculate conductive EF distributions within each model domain without considering transient behavior. Tetrahedral mesh elements were generated to discretize all geometries.

The in vitro model consisted of the ITO thin‐film electrode, culture medium, and an adherent cell layer positioned above the electrode surface (Figure S6a). The in vivo model consisted of the ITO thin‐film electrode, conductive hydrogel interface, and layered wound tissue, including epidermis, dermis, fat, and muscle. The wound was modeled as a circular defect with a diameter of 6 mm and a depth extending into the subcutaneous fat layer. The detailed three‐dimensional wound geometry is shown in Figure S7a,b.

For both models, the electrical properties of each domain were assumed to be homogeneous, linear, and isotropic. Thickness, conductivity, and relative permittivity values were assigned to each material or tissue domain based on literature values [75, 76, 77]. and were assumed to be spatially uniform and independent of the applied EF intensity. Electrode polarization and Faradaic reactions at the electrode‐electrolyte interface were not considered, and the analysis was limited to bulk conductive EF distributions. The electrical and geometrical parameters used in the in vitro and in vivo models are summarized in Table S3.

Boundary conditions were applied by assigning the stimulation voltage to the driving electrode and ground to the opposing electrode, while all remaining external boundaries were treated as electrically insulating. In the in vitro model, the EF magnitude was evaluated at 25 µm above the ITO surface. In the in vivo model, EF distributions were examined on a vertical cross‐section passing through the center of the wound model, and line profiles were extracted along the midline of each tissue layer. The applied voltage in the in vivo model was varied, and the resulting EF distributions were compared with those obtained from the in vitro model. The resulting EF distributions were visualized using surface and line plots.

### 4.12 Experimental Validation of EF at the Wound Tissue‐Device Interface

To experimentally validate the local EF predicted by FEA, voltage profiles were measured across the wound region under both in vivo and ex vivo conditions. A 5.00 V external potential was applied to the ITO‐based ES device using the same power supply configuration employed in the in vivo wound‐healing experiments. While the stimulation voltage was maintained, a fine‐tip probe connected to an oscilloscope (2 SERISE MSO, Tektronix, USA) was sequentially positioned at predefined locations spanning the wound boundary and adjacent skin regions to record the local electrical potential.

The local EF between two neighboring measurement positions was estimated from the spatial voltage gradient according to *EF = (V_n+1_‐V_n_)/d*. *d* is the distance between the corresponding measurement points (*d* = 2 mm). The calculated EF was assigned to the region between the two measurement positions to construct the experimental EF profile across the wound.

For the in vivo measurements, the ES device was conformally attached to a 6 mm dorsal skin wound using the same conductive hydrogel interface employed in the wound‐healing experiments, and local voltages were recorded while 5.00 V was applied through the device. For ex vivo validation, dorsal skin collected from a C57BL/6J mouse was prepared with a 6 mm circular wound matching the dimensions of the in vivo model. The wound cavity was filled with PBS to maintain a hydrated ionic environment at the tissue‐device interface, after which the conductive hydrogel and ES device were applied using the same configuration as in vivo. Voltage measurements and EF calculations were then performed at the corresponding positions using the identical 5.00 V stimulation condition. The resulting in vivo and ex vivo EF profiles were compared with the FEA‐predicted distribution to assess the experimental validity of the modeled electrical environment.

### 4.13 Ex Vivo Assessment of Electrical Stability during ES

Freshly excised mouse skin was rinsed with PBS and cut to an appropriate size for the ex vivo experiment. The skin samples were assembled with the ITO thin‐film device and conductive hydrogel in an ITO‐conductive hydrogel‐skin configuration. PBS was added to maintain tissue hydration and provide ionic contact throughout the experiment.

The ITO‐conductive hydrogel‐skin assembly was connected to a potentiostat (VSP‐300, BioLogic). Potentiostatic ES was applied at 5.00 V for 2 h, followed by a 1 h rest period without stimulation. This stimulation‐rest cycle was repeated eight times over a total period of 24 h. The current was monitored and recorded during each 2 h stimulation period to evaluate the electrical stability of the skin‐device assembly under repeated stimulation.

### 4.14 Measurement of pH at the Ex Vivo Skin‐Device Interface

Changes in pH at the ITO‐hydrogel‐skin interface were evaluated using DHPDS, a ratiometric fluorescent pH indicator previously used to monitor electrochemically induced pH changes [41, 42]. DHPDS was dissolved in PBS at a final concentration of 100 µM and was present in the solution throughout ES.

For calibration, PBS containing 100 µM DHPDS was adjusted to pH 5.0, 6.0, 7.0, and 8.0 using HCl or NaOH. The pH of each calibration solution was verified using a calibrated pH meter (Lab845, SI Analytics). Fluorescence intensities were measured using a microplate reader (Infinite M Plex, Tecan) at two excitation‐emission wavelength combinations: excitation at 410 nm and emission at 468 nm (F_468_), and excitation at 460 nm and emission at 498 nm (F_498_).

The ratiometric fluorescence response was calculated as:

$$R=\frac{F_{498}}{F_{468}}$$

A calibration curve was generated by plotting the fluorescence ratio against the corresponding pH of each calibration solution. All calibration standards and experimental samples were measured using identical instrumental settings and sample volumes.

Immediately after completion of each designated 2 h stimulation period, an aliquot of PBS was collected from the region adjacent to the ITO‐conductive hydrogel‐skin interface. Fluorescence intensities were measured under the same conditions used for calibration, and the pH of each sample was determined from the corresponding F_498_/F_468_ ratio using the calibration curve.

### 4.15 Measurement of H2O2 at the Ex Vivo Skin‐Device Interface

To assess ROS generation at the skin‐device interface during prolonged ES, H_2_O_2_ levels were quantified using an Amplex Red/HRP‐based fluorometric assay. An ex vivo wound model was prepared using mouse skin containing a 6 mm circular wound corresponding to that used in the in vivo experiments. The ITO‐based ES device and conductive hydrogel were applied to the wound region, and 5.00 V ES was delivered for 24 h using repeated 2 h ES/1 h rest cycles. Fluid samples were collected from the wound interface at defined time points throughout the stimulation period.

H_2_O_2_ was quantified using Amplex Red reagent and HRP (Invitrogen, USA). In the presence of HRP, H_2_O_2_ oxidizes Amplex Red to generate the fluorescent product resorufin, allowing H_2_O_2_ concentration to be determined from the resulting fluorescence signal. Collected wound‐interface samples were reacted with Amplex Red and HRP, and the resulting resorufin fluorescence was measured under identical conditions for all samples.

For quantitative calibration, H_2_O_2_ standard solutions ranging from 0.0 to 2.0 µm were prepared and analyzed using the same Amplex Red/HRP reaction conditions. The fluorescence intensity of resorufin was plotted against the corresponding H_2_O_2_ concentration and fitted by linear regression to generate a calibration curve. H_2_O_2_ concentrations in samples collected from the ex vivo wound interface were subsequently calculated from this calibration relationship and monitored throughout the 24 h cyclic ES period.

### 4.16 In Vivo Mouse Wound Healing Model

All animal procedures were performed in accordance with protocols approved by the Institutional Animal Care and Use Committee of the Korea Institute of Science and Technology (KIST‐IACUC‐2023‐048‐3). Seven‐week‐old male C57BL/6J mice were acclimated for 7 days before the experiments. For wound creation, mice were anesthetized with 2% isoflurane, and the dorsal skin was prepared by sequential hair removal using an electric clipper followed by depilatory cream treatment. The exposed skin was then disinfected with 70% ethanol. After gentle extension of the skin, a 6 mm circular area was marked using a biopsy punch, and the outlined tissue was excised with microscissors to create a full‐thickness excisional skin wound. Wound images were captured using an iPhone X camera (Model X, Apple, USA) at a fixed height under identical imaging conditions. The wound site was cleaned daily with 70% ethanol, and the device was replaced each day. At 7 days post‐wounding, the mice were euthanized, and skin tissue samples were collected for histological analysis. Wound area was quantified using ImageJ software, and values at each time point were normalized to the corresponding day 0 value. No animals were excluded from the analyses, and no data points were removed on the basis of experimental outcome.

### 4.17 Temperature Measurement

Temperature changes during ES were monitored using a thermal imaging camera (FLIR C3‐X, FLIR, USA). For the in vitro analysis, B16F10 cells were seeded at a density of 80,000 cells per well on a 1‐well culture slide containing an ITO thin‐film electrode. Samples were divided into non‐stimulated control and ES groups. In the ES group, 0.50 V was applied, and surface temperature was recorded every 1 h using thermal imaging. For the in vivo analysis, the dorsal hair of C57BL/6J mice was removed before device attachment. The ES device was then placed on the dorsal skin, and temperature changes at the skin‐device interface region were monitored over time during 5.00 V stimulation using a 2 h ES/1 h rest cycle, and a 5 h continuous stimulation condition. Thermal images were acquired during each stimulation condition to evaluate device‐associated temperature changes on the skin surface.

### 4.18 In Vivo ES Protocol

Conductive hydrogels (ST‐gel, Sekisuikasei, Japan) were cut into 2.5 cm × 1.5 cm, and a central 8 mm opening was created using a biopsy punch. Each hydrogel was assembled onto an ITO thin‐film‐based ES device, hydrated with saline, and positioned so that the wound was aligned within the central opening. The device was secured with a surgical bandage to minimize displacement during the treatment period. ES was delivered at 5.00 V in a cyclic regimen of 2 h ES followed by 1 h rest. For the ES + Nifedipine group, 10 µL of 50 µM nifedipine prepared in PBS was applied directly to the wound once daily immediately before attachment of the ES device. These animals received the same 5.00 V, 2 h ES/1 h rest stimulation regimen as the ES‐treated group. Following device application, mice were housed individually. For daily postoperative handling, the animals were briefly anesthetized with 2% isoflurane, the device was removed, the wound area and surrounding skin were cleaned, and a fresh device was applied.

### 4.19 Sample Collection and Tissue Preparation

Skin tissues from the wound area were excised with scissors, spread flat, and fixed overnight in 4% paraformaldehyde at 4°C. After tissue processing and paraffin embedding, the samples were sectioned at 5 µm using a microtome and mounted onto glass slides for subsequent analysis. Paraffin‐embedded skin tissue sections mounted on slides were dried in a 60°C oven for 30 min. The slides were then deparaffinized in xylene three times for 10 min each, followed by rehydration through a graded ethanol series (100%, 95%, 90%, 85%, 80%, and 70%) for 15 s each, and finally immersed in DW.

### 4.20 Histological Staining

For H&E staining, deparaffinized and rehydrated sections were stained using an H&E staining kit (ab245880, Abcam, UK). The sections were immersed in hematoxylin for 5 min, washed under running water for 5 min, and then immersed in bluing solution for 12 s. After two washes with DW, the slides were transferred to 100% ethanol, excess liquid was removed, and the sections were stained in eosin for 2 min and 30 s. The stained slides were then washed twice with 100% ethanol, air‐dried in a hood to remove residual ethanol, mounted with Permount Mounting Medium (SP15‐100, Fisher Chemical, UK), and coverslipped. MT staining was performed on deparaffinized and rehydrated sections using a modified protocol based on the manufacturer's instructions for the Masson's Trichrome Stain Kit (KH07007, Bioquochem, Spain). After excess DW was removed, the slides were incubated in Bouin's solution (BOU‐OT‐1L, BIOGNOST, Croatia) at 56°C for 1 h 30 min and then washed under running water for 20 min. The sections were subsequently immersed for 3 min in a 1:1 mixture of Weigert's hematoxylin reagents A and B, followed by washing under running water for 10 min. The slides were then immersed in Biebrich Scarlet solution for 12 min and washed three times with DW. The sections were incubated in phosphotungstic/phosphomolybdic acid solution for 15 min, and the residual solution was drained by tilting the slides before immersion in Aniline Blue solution for 15 min. The stained slides were then washed twice with 100% ethanol, dried, and mounted with Permount Mounting Medium (SP15‐100, Fisher Chemical, USA), followed by coverslipping.

### 4.21 Wound Length Measurement

Wound length was assessed from H&E‐stained cross‐sections of wound tissues harvested on day 7. Wound length was measured in ImageJ as the linear distance between the two opposing wound margins across the dermal wound bed. Measurements were obtained from five independent samples per group and used for quantitative comparison among the sham, device only, ES + Nifedipine, and ES groups.

### 4.22 IHC Staining

Following deparaffinization and rehydration, heat‐induced antigen retrieval was performed using EnVision FLEX Target Retrieval Solution, Low pH (50×) (K8005, Dako, USA) at 100°C for 15 min. The slides were then allowed to cool in a water bath and washed twice with DW. The sections were incubated in Tris‐buffered saline containing 0.05% Tween‐20 (TBST) for 15 min, followed by two additional washes with DW to remove residual Tween‐20. A hydrophobic barrier was drawn around each tissue section using an ImmEdge Hydrophobic Barrier PAP Pen (H‐4000, Vector Laboratories, USA). Primary antibodies were diluted 1:200 in Dako Antibody Diluent with Background Reducing Components (S3022, Dako, USA), and 200 µL of the antibody solution was applied to each section. The slides were incubated overnight at 4°C in a humidified chamber. The following day, the slides were washed three times for 10 min each with TBST. Secondary antibodies were diluted 1:200 in the same diluent, applied at 200 µL per section, and incubated for 1 h at room temperature in the dark. After three additional washes with TBST, the sections were mounted with 25 µL of VECTASHIELD HardSet Antifade Mounting Medium with DAPI (H‐1500‐10, Vector Laboratories, USA) and covered with coverslips. Details of the antibodies used are listed in Table S15.

### 4.23 Blood Vessel Quantification

Blood vessel‐like structures were assessed using H&E‐stained cross‐sections of wound tissues harvested on day 7. For each sample, images of the wound‐edge region were acquired under identical imaging conditions. Histologically identifiable blood vessel‐like structures were manually counted in ImageJ based on luminal morphology and the presence of erythrocytes. Quantitative values were acquired from five independent samples per group and used for statistical comparison among the sham, device only, ES + Nifedipine, and ES groups.

### 4.24 Proteomics by Nano LC‐MS/MS

HEMa cells were cultured individually under control conditions or subjected to 0.50 V ES for 48 h (n = 3 independent biological replicates per group), then harvested and washed with PBS. Cell pellets were lysed in SDT buffer containing 4% SDS, 2 mM Tris(2‐carboxyethyl) phosphine (TCEP, Sigma‐Aldrich, USA), and 0.1 M tetraethylammonium bromide (TEAB, Sigma‐Aldrich, USA) (pH 8.5), and denatured at 95°C for 15 min. After measuring protein concentration by the BCA assay, 90 µg of protein was prepared for digestion using the Suspension Trapping (S‐TRAP^TM^, PROTIFI, USA) filter micro spin column [78]. After loading the protein onto the Strap micro spin column, the samples were acidified and washed with phosphoric acid and wash buffer. Proteins were digested overnight at 37°C with sequencing‐grade trypsin (1:100, w/w, Promega, USA), and eluted three times with 50 mM TEAB, 0.2% formic acid, and 50% acetonitrile (ACN). Peptide concentrations were measured by BCA assay, and 10‐plex TMT labeling was performed by dissolving 0.8 mg TMT reagent in ACN and 70 µg of peptides for each channel. After 1 h incubation at room temperature, samples were quenched and acidified with 50 µL of 5% hydroxylamine and 20% formic acid. All Con and ES samples were labeled within a single TMT 10‐plex experiment and pooled before fractionation, therefore, no inter‐batch correction was required. Pooled samples were dried and desalted with reverse‐phase Oasis hydrophilic‐lipophilic balance (HLB) (3 cc). Labeled peptides were fractionated by high‐pH reverse‐phase chromatography into 12 fractions (5‐80% ACN in 15 mM ammonium hydroxide), lyophilized, and stored at −80°C. LC‐MS/MS was performed on a Q Exactive Plus mass spectrometer coupled to an Ultimate 3000 RSLC nano‐LC system with an EASY‐Spray source, using a trapping (75 µm × 150 mm, 3 µm) and analytical column (75 µm × 15 cm, 2 µm) with a 100 min nonlinear 8%–60% solvent B (100% ACN, 0.1% FA) gradient at 300 nL min^−1^. Full MS scans (m/z 300–1650) were acquired at 70,000 resolution (AGC 3 × 10^6^), followed by HCD MS/MS of the top 15 ions at 35,000 resolution (NCE = 32, isolation width = 1.2 m/z, AGC 2 × 10^5^, dynamic exclusion =  40 s). Raw data were processed in Proteome Discoverer 3.0 (UniProt Homo sapiens) with precursor tolerance of 10 ppm, fragment tolerance of 20 ppm, fixed modifications, TMT (N‐term, Lys) and carbamidomethyl‐Cys variable modifications, oxidation‐Met, and deamidation‐Asn/Gln. Protein abundances were normalized using the Total Peptide Amount normalization implemented in Proteome Discoverer. Peptide‐spectrum matches (PSMs) were filtered at 1% FDR (Percolator), and only spectra with complete reporter ion intensities across all channels were quantified. The normalized protein abundance data were subsequently analyzed using MetaboAnalyst 6.0 (https://www.metaboanalyst.ca/) for multivariate and exploratory differential‐abundance analyses [52]. Protein abundance values were mean‐centered prior to statistical analysis. Differences in protein abundance between the Con and ES groups were evaluated using an unpaired two‐tailed Student's t‐test in the Classical mode of MetaboAnalyst. Proteins with a nominal *p*‐value < 0.05 were considered candidates for exploratory analysis, and volcano plots were generated using a fold‐change threshold of 1.5 together with a nominal *p*‐value threshold of 0.05. SRplot (https://www.bioinformatics.com.cn/srplot) was used for functional annotation and visualization of enriched biological processes [79]. GO enrichment analysis was performed using the upregulated candidate protein set identified by a fold change > 1.5 and a nominal *p*‐value < 0.05. We evaluated GO‐term enrichment using a hypergeometric test, and we adjusted the resulting *p*‐values across the tested GO terms for multiple comparisons using the Benjamini‐Hochberg procedure. GO terms with an adjusted *p*‐value < 0.05 were considered significantly enriched.

### 4.25 Statistical Analysis

Data are presented as mean ± s.e.m. Statistical analyses were performed using Excel 16.111.2 and GraphPad Prism 10.3 for Windows. Comparisons were conducted using Student's t‐test, ANOVA, or multiple‐comparison tests, as appropriate for each experiment. For the 69‐gene RT‐qPCR panel, multiple testing correction was conducted using the two‐stage BKY procedure (FDR < 10%) to determine statistical significance. Statistical significance was defined as p < 0.05, with significance levels indicated as *p < 0.05, **p < 0.01, ***p < 0.001, and ****p < 0.0001.

## Author Contributions

Y.J.C., S.J.C., K.A.R., J.J., J.K.H., H.J., H.H., and J.P. designed all experiments and performed data analyses. Y.J.C. and K.A.R. performed in vitro analyses. T.K.L. conducted FEA simulations. G.L. and Y.J.C. performed ICC. Y.J.C., S.J.C., and K.A.R. performed in vivo wound healing experiments. H.L. and H.M. conducted proteomics analyses. Y.J.C., S.J.C., K.A.R., H.H., and J.P. wrote the manuscript.

## Conflicts of Interest

The authors declare no conflicts of interest.

## Supporting information




**Supporting File 1**: advs78525‐sup‐0001‐SuppMat.docx.

**Supporting File 2**: advs78525‐sup‐0002‐TableS1.xlsx.

**Supporting File 3**: advs78525‐sup‐0003‐TableS12.xlsx.

**Supporting File 4**: advs78525‐sup‐0004‐TableS13.xlsx.

**Supporting File 5**: advs78525‐sup‐0005‐TableS15.xlsx.

**Supporting File 6**: advs78525‐sup‐0006‐TableS17.xlsx.

## Data Availability

The data that support the findings of this study are available from the corresponding author upon reasonable request.
