# Supplementary material for: Adaptive Cellular Responsiveness to Electrical Stimulation at the Skin‐Electrode Interface
Source: Adv Sci (Weinh). 2026 Oct 27:e78525. Online ahead of print. doi: 10.1002/advs.78525 (PMC13712396; doi:10.1002/advs.78525)
Supplement: Supplementary file 1 [file ADVS-9999-e78525-s002.docx]

Supporting Information

**Adaptive Cellular Responsiveness to Electrical Stimulation**

**at the Skin-Electrode Interface**

Yeon Jae Chang, Soon Jin Choi, Khandoker Asiqur Rahaman, Tae Kyoung Lee, Gwangbin Lee, Jaeyeon Jun, Hyeon-Jeong Lee, Hophil Min, Hojeong Jeon, Jung Keun Hyun, Hyung-Seop Han*, Jimin Park*

Supporting Tables

**Table S1**

List of p-values of Figure 2a determined by t-test (attached to a separate Excel file).

**Table S2**

All p-values for Figure 2h were obtained by one-way ANOVA (F _(3, 8)_ = 92.60).

|  | **ES (-)**  **Nifedipine (-)** | **ES (-)**  **Nifedipine (+)** | **ES (+)**  **Nifedipine (-)** | **ES (+)**  **Nifedipine (+)** |
| --- | --- | --- | --- | --- |
| **ES (-)**  **Nifedipine (-)** | NA | 0.0009 | <0.0001 | 0.0883 |
| **ES (-)**  **Nifedipine (+)** | 0.0009 | NA | <0.0001 | 0.0281 |
| **ES (+)**  **Nifedipine (-)** | <0.0001 | <0.0001 | NA | <0.0001 |
| **ES (+)**  **Nifedipine (+)** | 0.0883 | 0.0281 | <0.0001 | NA |

**Table S3**

Electrical and geometrical parameters used for FEA (Figure 3b, d, Figure S6 and S7). Thickness, conductivity, and relative permittivity values for each domain in the in vitro and in vivo models.

| ***In vitro* model** | | | |
| --- | --- | --- | --- |
|  | Thickness (mm) | Conductivity (S/m) | Relative permittivity |
| ITO electrode | 0.10 | 1.67 $\times$ 10^5^ | 1 |
| Cell layer | 0.05 | 0.23 | 6000 |
| Culture medium | 5.00 | 1.68 | 80 |
| ***In vivo* model** | | | |
|  | Thickness (mm) | Conductivity (S/m) | Relative permittivity |
| ITO-based electrode | 0.10 | 1.67 $\times$ 10^5^ | 1 |
| Conductive hydrogel interface | 0.60 | 0.00017 | 80 |
| Epidermis | 0.05 | 0.12 | 6000 |
| Dermis | 0.50 | 0.23 | 6000 |
| Fat | 1.00 | 0.03 | 25000 |
| Muscle | 2.00 | 0.20 | 12500 |
| Blood | - | 0.70 | 80 |

**Table S4**

All p-values from one-way ANOVA (F _(3, 16)_ = 17.21) for Figure 3i.

|  | **Sham** | **Device only** | **ES** | **ES + Nifedipine** |
| --- | --- | --- | --- | --- |
| **Sham** | NA | 0.9862 | <0.0001 | 0.2761 |
| **Device only** | 0.9862 | NA | <0.0001 | 0.1611 |
| **ES** | <0.0001 | <0.0001 | NA | 0.0036 |
| **ES + Nifedipine** | 0.2761 | 0.1611 | 0.0036 | NA |

**Table S5**

Statistical comparison for Figure 3k using two-way ANOVA (Interaction F _(9, 64)_ = 4.276 (p-value = 0.0002), Row factor F _(3, 64)_ = 146.8 (p-value < 0.0001), Column factor F _(3, 64)_ = 28.11 (p-value <0.0001)).

| 0 day | **Sham** | **Device only** | **ES** | **ES + Nifedipine** |
| --- | --- | --- | --- | --- |
| **Sham** | NA | >0.9999 | >0.9999 | >0.9999 |
| **Device only** | >0.9999 | NA | >0.9999 | >0.9999 |
| **ES** | >0.9999 | >0.9999 | NA | >0.9999 |
| **ES + Nifedipine** | >0.9999 | >0.9999 | >0.9999 | NA |

| 3 day | **Sham** | **Device only** | **ES** | **ES + Nifedipine** |
| --- | --- | --- | --- | --- |
| **Sham** | NA | 0.2169 | 0.0005 | 0.2177 |
| **Device only** | 0.2169 | NA | <0.0001 | 0.0013 |
| **ES** | 0.0005 | <0.0001 | NA | 0.1188 |
| **ES + Nifedipine** | 0.2177 | 0.0013 | 0.1188 | NA |

| 5 day | **Sham** | **Device only** | **ES** | **ES + Nifedipine** |
| --- | --- | --- | --- | --- |
| **Sham** | NA | 0.6633 | <0.0001 | 0.7084 |
| **Device only** | 0.6633 | NA | <0.0001 | 0.1299 |
| **ES** | <0.0001 | <0.0001 | NA | 0.0001 |
| **ES + Nifedipine** | 0.7084 | 0.1299 | 0.0001 | NA |

| 7 day | **Sham** | **Device only** | **ES** | **ES + Nifedipine** |
| --- | --- | --- | --- | --- |
| **Sham** | NA | 0.5748 | <0.0001 | 0.2219 |
| **Device only** | 0.5748 | NA | 0.0023 | 0.9410 |
| **ES** | <0.0001 | 0.0023 | NA | 0.0162 |
| **ES + Nifedipine** | 0.2219 | 0.9410 | 0.0162 | NA |

**Table S6**

One-way ANOVA (F _(3, 16)_ = 26.56) derived p-values for Figure 3m.

|  | **Sham** | **Device only** | **ES** | **ES + Nifedipine** |
| --- | --- | --- | --- | --- |
| **Sham** | NA | 0.0327 | <0.0001 | 0.3839 |
| **Device only** | 0.0327 | NA | 0.0129 | 0.0012 |
| **ES** | <0.0001 | 0.0129 | NA | <0.0001 |
| **ES + Nifedipine** | 0.3839 | 0.0012 | <0.0001 | NA |

**Table S7**

All p-values of Figure 4c by one-way ANOVA (F _(3, 16)_ = 50.82).

|  | **Sham** | **Device only** | **ES** | **ES + Nifedipine** |
| --- | --- | --- | --- | --- |
| **Sham** | NA | 0.8814 | <0.0001 | 0.5574 |
| **Device only** | 0.8814 | NA | <0.0001 | 0.9318 |
| **ES** | <0.0001 | <0.0001 | NA | <0.0001 |
| **ES + Nifedipine** | 0.5574 | 0.9318 | <0.0001 | NA |

**Table S8**

Figure 4e p-values determined from one-way ANOVA (F _(3, 16)_ = 19.77).

|  | **Sham** | **Device only** | **ES** | **ES + Nifedipine** |
| --- | --- | --- | --- | --- |
| **Sham** | NA | 0.9615 | <0.0001 | 0.9863 |
| **Device only** | 0.9615 | NA | 0.0001 | 0.8434 |
| **ES** | <0.0001 | 0.0001 | NA | <0.0001 |
| **ES + Nifedipine** | 0.9863 | 0.8434 | <0.0001 | NA |

**Table S9**

All p-values of Figure 4g by one-way ANOVA (F _(3, 16)_ = 91.60).

|  | **Sham** | **Device only** | **ES** | **ES + Nifedipine** |
| --- | --- | --- | --- | --- |
| **Sham** | NA | 0.5429 | <0.0001 | 0.8853 |
| **Device only** | 0.5429 | NA | <0.0001 | 0.2009 |
| **ES** | <0.0001 | <0.0001 | NA | <0.0001 |
| **ES + Nifedipine** | 0.8853 | 0.2009 | <0.0001 | NA |

**Table S10**

P-values of Figure 5b determined by t-test.

| Gene | *p*-values | t ratio | Degree of freedom |
| --- | --- | --- | --- |
| *ERK* | 0.004205 | 5.871 | 4.000 |
| *MEK* | 0.000832 | 9.033 | 4.000 |
| *PI3K* | 0.001956 | 7.216 | 4.000 |
| *Raf* | 0.023243 | 3.576 | 4.000 |
| *AKT* | 0.018876 | 3.814 | 4.000 |
| *CACNA1D* | 0.013587 | 4.210 | 4.000 |
| *mTOR* | 0.034538 | 3.149 | 4.000 |
| *GSK3β* | 0.008916 | 4.758 | 4.000 |

**Table S11**

All p-values for Figure 5e were obtained by one-way ANOVA (F _(3, 12)_ = 113.5).

|  | **ES (-)**  **Nifedipine (-)** | **ES (-)**  **Nifedipine (+)** | **ES (+)**  **Nifedipine (-)** | **ES (+)**  **Nifedipine (+)** |
| --- | --- | --- | --- | --- |
| **ES (-)**  **Nifedipine (-)** | NA | <0.0001 | <0.0001 | 0.9969 |
| **ES (-)**  **Nifedipine (+)** | <0.0001 | NA | <0.0001 | <0.0001 |
| **ES (+)**  **Nifedipine (-)** | <0.0001 | <0.0001 | NA | <0.0001 |
| **ES (+)**  **Nifedipine (+)** | 0.9969 | <0.0001 | <0.0001 | NA |

**Table S12**

P-values for Figure 5f were determined by t-test (provided in a separate Excel file).

**Table S13**

List of primers used in B16F10 qPCR (attached to a separate Excel file).

**Table S14**

Primer list used for HEMa qPCR.

| Gene | Primer forward | Primer reverse |
| --- | --- | --- |
| *GAPDH* | CACCAGGGCTGCTTTTAACTCT | CTTCCCGTTCTCAGCCTTGACC |
| *CACNA1D* | CTTCGACAACGTCCTCTCTGCT | GCCGATGTTCTCTCCATTCGAG |
| *AKT* | ACTCATTCCAGACCCACGAC | AGCCCGAAGTCCGTTATCTT |
| *mTOR* | ATGCTTGGAACCGGACCTG | TCTTGACTCATCTCTCGGAGTT |
| *GSK3β* | CCGACTAACACCACTGGAAGCT | AGGATGGTAGCCAGAGGTGGAT |
| *ERK* | GAACTCCAAGGGCTATACCAAGT | GGAGGGCAGAGACTGTAGGTAGT |
| *PI3K* | GGCTTGGACCGAATGCT | TTGTTGAAGGCTGTGGC |
| *Raf* | CGAACAGTGAATATTTCCTTTGAT | TCCCTCTCAGGCATAAGGTAA |
| *MEK* | GCTTCTATGGTGCGTTCT | GAGTTGACTAGGATGTTGGA |

**Table S15**

List of antibodies used in this paper (attached to a separate Excel file).

**Table S16**

All p-values of Figure S1b by one-way ANOVA (F _(4, 10)_ = 5.029)

|  | **Con** | **0.25 V** | **0.50 V** | **0.75 V** | **1.00 V** |
| --- | --- | --- | --- | --- | --- |
| **Con** | NA | 0.9987 | 0.0365 | > 0.9999 | > 0.9999 |
| **0.25 V** | 0.9987 | NA | 0.0242 | 0.9981 | 0.9965 |
| **0.50 V** | 0.0365 | 0.0242 | NA | 0.0381 | 0.0413 |
| **0.75 V** | > 0.9999 | 0.9981 | 0.0381 | NA | > 0.9999 |
| **1.00 V** | > 0.9999 | 0.9965 | 0.0413 | > 0.9999 | NA |

**Table S17**

List of p-values and false positive threshold values of Figure S2 determined by two-stage BKY multiple comparison (attached to a separate Excel file).

**Table S18**

All p-values of Figure S11b were obtained by two-way ANOVA (Interaction F _(6, 24)_ = 1.487 (p-value = 0.2249), Row factor F _(3, 24)_ = 79.18 (p-value < 0.0001), Column factor F _(2,24)_ = 7.446 (p-value = 0.0030)).

| 0 day | **Sham** | **Device only** | **ES** |
| --- | --- | --- | --- |
| **Sham** | NA | >0.9999 | >0.9999 |
| **Device only** | >0.9999 | NA | >0.9999 |
| **ES** | >0.9999 | >0.9999 | NA |

| 3 day | **Sham** | **Device only** | **ES** |
| --- | --- | --- | --- |
| **Sham** | NA | 0.9316 | 0.1189 |
| **Device only** | 0.9316 | NA | 0.0584 |
| **ES** | 0.1189 | 0.0584 | NA |

| 5 day | **Sham** | **Device only** | **ES** |
| --- | --- | --- | --- |
| **Sham** | NA | 0.9974 | 0.0113 |
| **Device only** | 0.9974 | NA | 0.0132 |
| **ES** | 0.0113 | 0.0132 | NA |

| 7 day | **Sham** | **Device only** | **ES** |
| --- | --- | --- | --- |
| **Sham** | NA | 0.3782 | 0.1528 |
| **Device only** | 0.3782 | NA | 0.8376 |
| **ES** | 0.1528 | 0.8376 | NA |

**Table S19**

All p-values of Figure S12 obtained by one-way ANOVA (F _(2, 6)_ = 1.782).

|  | **Sham** | **Device only** | **ES** |
| --- | --- | --- | --- |
| **Sham** | NA | 0.7613 | 0.5208 |
| **Device only** | 0.7613 | NA | 0.2267 |
| **ES** | 0.5208 | 0.2267 | NA |

**Table S20**

All p-values of Figure S17 by one-way ANOVA (F _(2, 12)_ = 73.71).

|  | **Sham** | **Device only** | **ES** |
| --- | --- | --- | --- |
| **Sham** | NA | 0.9793 | <0.0001 |
| **Device only** | 0.9793 | NA | <0.0001 |
| **ES** | <0.0001 | <0.0001 | NA |

**Table S21**

All p-values of Figure S18 by one-way ANOVA (F _(3, 16)_ = 30.04).

|  | **Sham** | **Device only** | **ES** | **ES + Nifedipine** |
| --- | --- | --- | --- | --- |
| **Sham** | NA | 0.0261 | 0.0001 | 0.00326 |
| **Device only** | 0.0261 | NA | 0.0729 | <0.0001 |
| **ES** | 0.0001 | 0.0729 | NA | <0.0001 |
| **ES + Nifedipine** | 0.00326 | <0.0001 | <0.0001 | NA |

**Table S22**

Summary of all p-values of Figure S19 based on one-way ANOVA (F _(3, 16)_ = 13.22).

|  | **Sham** | **Device only** | **ES** | **ES + Nifedipine** |
| --- | --- | --- | --- | --- |
| **Sham** | NA | 0.5669 | 0.0001 | 0.3490 |
| **Device only** | 0.5669 | NA | 0.0014 | 0.9778 |
| **ES** | 0.0001 | 0.0014 | NA | 0.0032 |
| **ES + Nifedipine** | 0.3490 | 0.9778 | 0.0032 | NA |

**Table S23**

One-way ANOVA (F _(2, 12)_ = 123.0) p-values reported for Figure S20.

|  | **Sham** | **Device only** | **ES** |
| --- | --- | --- | --- |
| **Sham** | NA | 0.8426 | <0.0001 |
| **Device only** | 0.8426 | NA | <0.0001 |
| **ES** | <0.0001 | <0.0001 | NA |

**Table S24**

All p-values for Figure S22 were obtained by one-way ANOVA (F _(3, 16)_ = 0.3477).

|  | **Sham** | **Device only** | **ES** | **ES + Nifedipine** |
| --- | --- | --- | --- | --- |
| **Sham** | NA | >0.9999 | 0.8839 | 0.8904 |
| **Device only** | >0.9999 | NA | 0.8832 | 0.8898 |
| **ES** | 0.8839 | 0.8832 | NA | >0.9999 |
| **ES + Nifedipine** | 0.8904 | 0.8898 | >0.9999 | NA |

**Table S25**

One-way ANOVA (F _(3, 16)_ = 992.9) p-values reported for Figure S24.

|  | **Sham** | **Device only** | **ES** | **ES + Nifedipine** |
| --- | --- | --- | --- | --- |
| **Sham** | NA | 0.9399 | <0.0001 | 0.9910 |
| **Device only** | 0.9399 | NA | <0.0001 | 0.8248 |
| **ES** | <0.0001 | <0.0001 | NA | <0.0001 |
| **ES + Nifedipine** | 0.9910 | 0.8248 | <0.0001 | NA |

**Table S26**

All p-values of Figure S25b cell proliferation by one-way ANOVA (F _(3, 8)_ = 122.7)

|  | **Con** | **0.25 V** | **0.50 V** | **0.75 V** |
| --- | --- | --- | --- | --- |
| **Con** | NA | 0.2071 | <0.0001 | 0.0171 |
| **0.25 V** | 0.2071 | NA | <0.0001 | 0.3406 |
| **0.50 V** | <0.0001 | <0.0001 | NA | <0.0001 |
| **0.75 V** | 0.0171 | 0.3406 | <0.0001 | NA |

**Figure S1** **Viability and proliferation of B16F10 cells under various ES conditions. (a)** Representative immunofluorescence images of live and dead analysis of B16F10 cells cultured on the ITO thin-film culture platform (scale bar = 100 μm, voltage range = 0.00-1.00 V). **(b)** Cell proliferation was measured with CCK-8 analysis under these voltage conditions. Data in Figure S1b were analyzed by one-way ANOVA and Tukey’s multiple comparison test (*n* = 3, all p-values are listed in **Table S16**). Data are presented as mean ± s.e.m. Statistical significance indicated as *p < 0.05.

**Figure S2 Two-stage Benjamini-Krieger-Yekutieli (BKY) multiple testing correction of 69-gene qPCR panel.** Raw p-values (purple line) were ranked in ascending order and compared with the BKY false positive threshold (black line) calculated using the two-stage BKY procedure at FDR < 10%. Of the 19 genes showing nominal significance before correction (p < 0.05), 18 remained significant after FDR correction. *KCNA10*, a potassium channel-related gene, did not meet the BKY false positive threshold and was excluded from the final set of significant genes. The right panel shows an enlarged view of the 18 FDR-significant genes (all p-values and false positive threshold are listed in **Table S17**).

**Figure S3 Enhanced Ca_V_1.3 expression in HUVECs following ES. (a)** Representative HUVECs ICC images of Ca_V_1.3 expression (green) and DAPI-stained nuclei (blue) in Con and 0.25 V ES after 48 h (scale bar, 200 μm). **(b)** Quantification of Ca_V_1.3 fluorescence intensity normalized to the number of DAPI-positive cells (Ca_V_1.3/DAPI count) demonstrated a significant increase in the ES group compared with the Con group (*n* = 3 per group, p-value = 0.0011, t = 8.424, df = 4). **p < 0.01.

**Figure S4** **Ca_V_1.3 upregulation in NIH3T3 cells under ES. (a)** Representative ICC images of NIH3T3 cells maintained under control conditions or exposed to ES at 0.50 V for 24 h. Ca_V_1.3 is shown in green, and nuclei are counterstained with DAPI in blue (scale bar, 200 μm). **(b)** Ca_V_1.3 fluorescence normalized to the DAPI-positive cell count increased in electrically stimulated NIH3T3 cells relative to Con (*n* = 3 per group; p = 0.0008, t = 9.076, df = 4). *******p < 0.001.

**Figure S5 Effects of nifedipine on cell proliferation upon ES.** Cell proliferation was assessed by CCK-8 assay under 0.50 V ES conditions with or without nifedipine treatment (10 μM). Nifedipine significantly reduced cell proliferation in ES-pretreated cells. Data are presented as mean ± s.e.m. (*n* = 3 per group, p-value = 0.0049, t = 5.641, df = 4). Statistical significance was determined using a t-test. **p < 0.01.

**Figure S6 Geometry of the in vitro ES model used for FEA and calculated EF profile.** **(a)** A schematic illustration of the ITO thin-film culture platform configuration with cells for FEA analysis. **(b)** Calculated cross-sectional EF distribution in the configuration shown in **(a)** under an applied voltage of 0.50 V. **(c)** Calculated EF profile extracted at 25 μm above the ITO thin-film electrode surface. All parameters used in the FEA simulation are listed in **Table S3**.

**Figure S7 Geometry of the in vivo wound model used for FEA and calculated EF profiles of fat and muscle layers.** **(a)** 3D model of the in vivo wound configuration. **(b)** Cross-sectional view of the in vivo wound model, showing the positions of the tissue regions used for EF analysis (all parameters that were used in the FEA simulation are listed in **Table S3)**. **(c)** Calculated in-plane EF maps of fat and muscle layers, under an applied voltage of 5.00 V. **(d)** Quantitative EF profile along the lateral axis in the fat and muscle layers, showing EF peaks near the wound margins under the device.

**Figure S8 Experimental measurement of EF distribution across the wound region.** **(a)** Schematic illustration of the experimental setup for measuring the local EF at the skin tissue-device interface under an applied voltage of 5.00 V. Local voltages were measured at defined positions using a fine-tip probe connected to an oscilloscope, and the EF between adjacent positions was estimated from the measured voltage difference and corresponding distance. **(b)** Experimentally measured EF profiles across the wound region under in vivo (magenta line) and ex vivo (blue line) conditions. Orange dashed lines indicate the wound boundaries, and the orange-shaded region represents the wound area. The measured EF profiles showed comparable distributions between the in vivo and ex vivo conditions.

**Figure S9 Temperature measurement of the in vitro platform during continuous ES. (a)** Representative thermal images of the in vitro ITO thin-film platform under non-stimulated control group (Con) and 0.50 V ES-treated group (ES) after 24 h. Thermal images were acquired during cell culture to evaluate temperature changes at the device-contact region (scale bar = 2 cm). **(b)** Time-dependent temperature profiles of the in vitro platform under Con and ES for 24 h. No significant temperature difference was observed between the Con and ES groups.

**Figure S10 Temperature change of the in vivo ITO thin-film-based ES device under different ES durations.** Representative thermal images of an ITO thin-film-based ES device (white dashed box) attached to the dorsal skin of a mouse with 5.00 V ES. Thermal imaging showed that the 2 h ES/1 h rest cycle did not significantly alter the local temperature (ΔT < 1.5 °C), whereas continuous 5 h stimulation increased the local temperature to > 4 °C (scale bar = 2 cm).

**Figure S11 Wound closure analysis under 1 h ES/1 h rest cycles.** (**a**) Representative serial photographs of wound regions in the sham, device only, and ES groups at days 0, 3, 5, and 7 after wound creation (scale bar = 5 mm). (**b**) Quantitative analysis of wound closure over 7 days under the 1 h ES/1 h rest regimen. Statistical analysis was performed using two-way ANOVA followed by Tukey’s multiple comparison test (n = 3 per group, all p-values are listed in **Table S18**). Data are presented as mean ± s.e.m. Statistical significance indicated as *p < 0.05, **p < 0.01.

**Figure S12 Ca_V_1.3 expression in wound tissue under a 1 h ES/1 h rest stimulation regimen.** (**a**) Representative immunofluorescence images of day 7 wound tissue sections from the sham, device only, and ES groups following the 1 h ES/1 h rest regimen, showing Ca_V_1.3 (green) and DAPI (blue) staining. Light-blue dashed lines indicate the wound boundaries (scale bar = 400 μm). (**b**) Quantification of Ca_V_1.3 fluorescence intensity normalized to DAPI count in the wound tissue sections. Statistical analysis was performed using one-way ANOVA followed by Tukey’s multiple comparison test (n = 3 per group, all p-values are listed in **Table S19**). Data are presented as mean ± s.e.m.

**Figure S13 Electrical stability at the ex vivo skin-device interface under cyclic ES.** Current profile measured over 24 h in an ex vivo wound model subjected to 5.00 V ES using repeated 2 h ES/1 h rest cycles. The sky-blue line represents the measured current during ES, while the pink shaded regions indicate the ES periods separated by 1 h rest intervals. The current remained relatively stable throughout repeated stimulation cycles, indicating consistent electrical delivery over the 24 h device-use period.

**Figure S14 DHPDS-based ratiometric pH measurement at the ex vivo skin-device interface under cyclic ES.** **(a)** Schematic illustration of pH monitoring at the ex vivo wound skin-device interface during 24 h stimulation using the same 5.00 V, 2 h ES/1 h rest regimen employed in the in vivo experiments. Local pH was determined using the ratiometric fluorescent photoacid dye DHPDS by measuring its the ratio of fluorescence emission intensities. (**b**) Calibration curve relating the ratiometric fluorescence response of DHPDS to pH. Linear regression showed a strong correlation between the ratio of fluorescence emission intensities and pH (R^2^ = 0.9925), which was used to determine the pH of samples collected from the ex vivo wound region during ES.

**Figure S15 Amplex Red/HRP-based H_2_O_2_ measurement at the ex vivo skin tissue-device interface under 2 h ES/1 h rest cyclic stimulation.** (**a**) Experimental scheme for assessing H_2_O_2_ production in an ex vivo wound model subjected to 5.00 V ES using the 2 h ES/1 h rest cycle for 24 h. Samples were collected from the wound region and analyzed with the Amplex Red and HRP fluorometric assay. In this reaction, H_2_O_2_-dependent oxidation of Amplex Red by HRP produces fluorescent resorufin, allowing quantitative determination of H_2_O_2_ generated at the wound tissue-device interface. (**b**) Standard calibration curve obtained from known H_2_O_2_ concentrations and the corresponding resorufin fluorescence intensities (R^2^ = 0.9998). The resulting linear relationship was used to calculate H_2_O_2_ concentrations in samples collected during ex vivo ES.

**Figure S16 Histological analysis of VGCC expression and vascular development.** Representative single-channel immunofluorescence images of wound tissue sections collected on day 7 from sham, device only, ES + Nifedipine, and ES groups stained for Ca_V_1.3 (green), CD31 (red), and DAPI (blue). The wound regions are shown in light-blue dashed lines (scale bar = 400 μm).

**Figure S17 Co-localization analysis of Ca_V_1.3 and α-SMA in wound tissue.** (**a**) Representative immunofluorescence images of day 7 wound tissue sections from the sham, device only, and ES groups, displaying individual and merged signals for Ca_V_1.3 (green), α-SMA (red), and DAPI (blue). The light-blue dashed lines delineate the wound boundaries (scale bar = 400 μm). (**b**) Quantification of the Ca_V_1.3 and α-SMA co-localized area within the wound region. The ES group showed a significantly greater co-localized area than the sham and device only groups. Statistical analysis was performed using one-way ANOVA followed by Tukey’s multiple comparison test (n = 5 per group, all p-values are listed in **Table S20**). Data are presented as mean ± s.e.m. Statistical significance indicated as ****p < 0.0001.

**Figure S18 Wound length analysis with H&E staining. (a)** Representative cross-sectional H&E histological images of mouse skin tissue harvested from sham, device only, ES + Nifedipine, and ES groups after 7 days. Yellow dashed lines represent the wound region of these samples (scale bar = 500 μm). **(b)** Quantitative comparison of wound length of these groups, analyzed by one-way ANOVA and Tukey’s multiple comparison test (*n* = 5 per group, all p-values are listed in **Table S21**). Data are presented as mean ± s.e.m. Statistical significance is indicated as ****p < 0.0001.

**Figure S19 Blood vessel counting analysis with H&E staining.** (**a**) Representative H&E histological images of wound tissue sections from sham, device only, ES + Nifedipine, and ES groups, with yellow circles representing blood vessels (scale bar = 500 μm). (**b**) Quantification of blood vessel counts from H&E histological sections (n = 5 per group, all p-values are listed in **Table S22**). Statistical significance is indicated as ***p < 0.001.

**Figure S20 Quantitative co-localization of CaV1.3 and CD31 in wound tissue.** (**a**) Representative merged immunofluorescence images of day 7 wound sections from sham, device only, and ES groups, showing Ca_V_1.3 (green) and CD31 (red) signals within the wound region. The light-blue dashed lines delineate the wound area, and the corresponding single-channel images are provided in Figure S16 (scale bar = 400 μm). (**b**) Quantitative comparison of the Ca_V_1.3 and CD31 co-localized area within the wound region. Statistical analysis was performed using one-way ANOVA followed by Tukey’s multiple comparison test (n = 5 per group, all p-values are listed in **Table S23**). Data are presented as mean ± s.e.m. Statistical significance indicated as ****p < 0.0001.

**Figure S21 Immunofluorescence analysis of CD68 and CD206 expression in wound tissue sections.** Single- and merged-channel immunofluorescence images showing CD68 (green), CD206 (magenta), and DAPI (blue) staining in day 7 wound regions from sham, device only, ES + Nifedipine, and ES groups. The wound regions are marked with a light-blue dashed line (scale bar = 400 μm).

**Figure S22 Quantification of macrophages using CD68 expression in wound tissue sections.** Quantification of normalized CD68 intensity relative to DAPI count (n = 5 per group, all single-channel and merged-channel images are displayed in Figure S21). Data are presented as mean ± s.e.m., and statistical analyses were performed using one-way ANOVA followed by Tukey’s multiple-comparison test. All p-values are listed in **Table S24**.

**Figure S23 Immunofluorescence analysis of CD68 and iNOS in wound tissue.** Representative single-channel and merged immunofluorescence images of day 7 wound tissue sections from sham, device only, ES + Nifedipine, and ES groups stained for CD68 (green), iNOS (red), and DAPI (blue) (scale bar = 400 μm). A light-blue dashed line represents the wound region.

**Figure S24 Quantification of CD206^+^/iNOS^+^ ratio in wound tissue sections.** Quantification of CD206^+^/iNOS^+^ ratio, calculated from the data in Figure 4e and Figure 4g (n = 5 per group). All single-channel and merged-channel images are displayed in Figure S21 (CD206) and Figure S23 (iNOS). Data are presented as mean ± s.e.m., and statistical analyses were performed using one-way ANOVA followed by Tukey’s multiple-comparison test. Statistical significance is indicated as ****p < 0.0001. All p-values are listed in **Table S25**.

**Figure S25 HEMa proliferation under different ES conditions.** **(a)** Representative immunofluorescence images of live and dead analysis of HEMa cultured on an ITO thin-film platform under different ES conditions (scale bar = 200 μm, voltage range = 0.00-0.75 V). **(b)** Cell proliferation under each voltage condition was quantified using the CCK-8 assay. Data were analyzed by one-way ANOVA and Tukey’s multiple comparison test (*n* = 3, all p-values are listed in **Table S26**). Data are presented as mean ± s.e.m. Statistical significance indicated as *p < 0.05, ****p < 0.0001.

**Figure S26 HEMa cell proliferation under ES and ES with nifedipine treatment.** HEMa cell proliferation was quantified by CCK-8 assay after 48 h of 0.50 V ES with or without nifedipine treatment (10 μM). Nifedipine treatment significantly reduced cell proliferation compared with ES alone. Data are presented as mean ± s.e.m. (*n* = 3 per group, p-value < 0.0001, t = 20.89, df = 4). Statistical significance was determined using a t-test. ****p < 0.0001.

**Figure S27** **Principal component analysis (PCA) of proteomic profiles in control and ES-treated (0.50 V) human skin cells.** Scores plot showing clear separation between groups along PC1 (65.4%) and PC2 (15.2%), indicating distinct proteomic signatures induced by ES (*n* = 3 independent biological replicates).

**Figure S28 List of proteins upregulated in human skin cells treated with ES and their functional roles.** The 11 proteins with the highest FC values among ES-upregulated proteins were selected from proteomic analysis and categorized according to representative biological functions. The annotated proteins are associated with transcriptional and post-transcriptional regulation, ribosome biogenesis, mitochondrial metabolism, and cytoskeletal or structural remodeling. These functional annotations provide additional context for the ES-induced proteomic remodeling observed in HEMa cells (*n* = 3 independent biological replicates).

**Figure S29 GO-chord plot of protein-function associations in ES-treated HEMa cells.** The associations between differentially expressed proteins (left) and enriched functional categories (right) in ES-treated human skin cells are illustrated in a GO-chord plot. Colored ribbons indicate protein contributions to biological processes, whereas segment colors signify functional groups including cell organization, metabolism, signal transduction, and stress response. The intensity of protein color correlates with the log FC (*n* = 3 independent biological replicates).
